# Spider Toxin SNX-482 Gating Modifier Spontaneously Partitions in the Membrane Guided by Electrostatic Interactions

**DOI:** 10.3390/membranes12060595

**Published:** 2022-06-06

**Authors:** Guido Mellado, Nicolas Espinoza, Jose Antonio Garate, Alan Neely

**Affiliations:** 1 Centro Interdisciplinario de Neurociencias de Valparaíso, Facultad de Ciencias, Universidad de Valparaiso, Valparaiso 2362735, Chile; guido.mellado@cinv.cl (G.M.); nicolas.espinozamu@cinv.cl (N.E.); 2 Biofisica y Biología Computacional, Facultad de Ciencias, Universidad de Valparaiso, Valparaiso 2362735, Chile; 3 Millennium Nucleus in NanoBioPhysics, Facultad de Ciencias, Universidad de Valparaiso, Valparaiso 2360102, Chile; 4 Centro Científico y Tecnológico de Excelencia Ciencia y Vida, Santiago 7750000, Chile

**Keywords:** calcium channel, gating modifier, SNX-482, cysteine-rich peptide, membrane partitioning

## Abstract

Spider toxin SNX-482 is a cysteine-rich peptide that interferes with calcium channel activity by binding to voltage-sensing domains of the Ca_V_2.3 subtype. Two mechanisms dominate the binding process of cysteine-rich peptides: direct binding from the aqueous phase or through lateral diffusion from the membrane, the so-called reduction in dimensionality mechanism. In this work, via coarse-grained and atomistic molecular dynamics simulations, we have systematically studied the spontaneous partitioning of SNX-482 with membranes of different anionic compositions and explored via diffusional analysis both binding mechanisms. Our simulations revealed a conserved protein patch that inserts in the membrane, a preference for binding towards partially negatively charged membranes, and that electrostatics guides membrane binding by incrementing and aligning the molecular dipole. Finally, diffusivity calculations showed that the toxin diffusion along the membrane plane is an order of magnitude slower than the aqueous phase suggesting that the critical factor in determining the SNX-482-Ca_V_2.3 binding mechanism is the affinity between the membrane and SNX-482.

## 1. Introduction

Among animal species that use poison to kill or paralyze prey, spiders emerge as the richest natural sources of toxic peptides. This group of peptides can be classified based on their number of disulfide bonds that range from one to seven [1]. The most abundant group of spider toxins share a structural pattern called inhibitor cystine knot (ICK), characterized by disulfide bridges. ICK structural motif is defined by an antiparallel beta-sheet stabilized by a ring formed by two disulfide bridges (Cys1-Cys4 and Cys2-Cys5) and a disulfide bond (Cys3-Cys6) that passes through the ring [2,3]. These internal bridges confer exceptional resistance to high temperatures (80 °C), protease degradation, extreme pH, and organic solvents [4]. ICKs preferred targets are voltage-dependent ion channels [5,6]. Channels from the superfamily of voltage-gated channels are endowed with four voltage-sensing domains (VSD) surrounding a single pore domain (PD) at the center of symmetry. Each VSD consists of four transmembrane helices flanked by two intracellular segments. The C-terminal end of each VSD connects to two transmembrane segments contributing to the PD. ICK toxins modulate voltage-gated channel activity by two mechanisms: preventing ion flow by occluding the channel as is the case of charybdotoxin inhibiting Ca^+2^ activated K+ channels called pore-blocker [7]; or interfering with voltage-dependent gating such as hanatoxin (HaTx) and VSTx1 [8,9] named gating modifiers toxins (GMTx). In contrast to pore-blockers that diffuse through the aqueous phase, GMTx meander along the membrane plane to reach their target. Most spider GMTx had adopted the “reduction-of-dimensionality” (RD) strategy [10] that is preceded by an abrupt increase in local concentration mediated by non-specific binding to the outer leaflet of the membrane. The formation of the ternary complex with the channel protein is thought to be favored by reactants diffusion in two dimensions. However, there is no direct evidence supporting this view. Membrane partition may also increase the local concentration dramatically. The question then arises to what extent the apparent binding affinity measured experimentally is dominated by the significant free energy change (ΔG) of the toxin partitioning into the membrane [11,12]. How does the membrane lipid composition contribute to this process? Previous studies have established a preference for membranes rich in anionic phospholipids [13,14]. However, the requirement for anionic lipids for membrane partitioning remains controversial since GMTx with a high affinity to neutral or mixed membrane models have been identified. [8,12,15]. Since most studied GMTx are positively charged, it is tempting to conclude that the net charge is relevant to toxin-membrane interaction. Still, membrane-access mechanisms cannot be generalized to all GMTx, and they must be investigated case-by-case. An interesting example is SNX-482, which is unique in having a negative net charge of minus two. SNX-482 is a 41 residue GMTx isolated from African tarantula venom, *Hysterocrates gigas* [16], and is the only reported potent antagonist of the neuronal calcium channel subtype Ca_V_2.3 [17]. SNX-482 inhibits activation and is thought to interact with VSD of repeats three and four of Ca_V_2.3 channels. The binding of SNX-482 to the VSD would hinder its activation, shifting voltage dependence by about +60 mV. VSD-Toxin interaction can be reversed by strong depolarization, an observation predicted by a model in which SNX-482 prefers the resting state of the VSD [18]. The enthusiasm for SNX-482 as a therapeutic agent was somewhat diminished by discovering that it also blocks potassium channels Kv4.2 and Kv4.3 [19]. However, recent research could position SNX-482 as an interesting molecule for anticancer immunotherapy [20].

This work extensively investigates the spontaneous partitioning of SNX-482 into lipid bilayers of different anionic compositions using coarse-grained (CG) and all-atom (AT) molecular dynamics simulations. As a whole, our simulations consistently revealed a conserved protein patch that inserts within the membrane. Furthermore, atomistic simulations exhibited a preference for partially negatively charged bilayers. Despite a net charge of minus two, free energy and macrodipole calculations indicate that dispersion interactions energetically drive binding to the membrane and that electrostatics guide proper dipole alignment and configuration for membrane adsorption. Finally, diffusivity calculations showed that the toxin diffusion along the membrane plane is at least an order of magnitude slower than in the aqueous phase, suggesting that reducing dimensionality does not aid SNX-482-Ca_V_2.3 binding. However, slow membrane diffusion can compensate for a reaction-limited binding by increasing the local concentration of the ligand as expressed in the partition coefficient [10]. Thus, increasing membrane partitioning and the ensuing increase in local concentration reduces the effective toxin concentration in solution, relaxing the structural requirement for tight binding to the receptor.

## 2. Materials and Methods

### 2.1. SNX-482 Modeling

**Figure 1:**
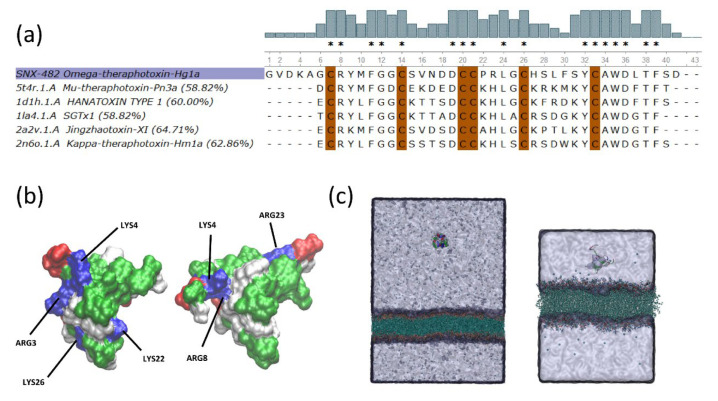
Comparative Modeling SNX-482. (**a**) Alignments of the five ICK toxins with the highest identity score to SNX-482. Identity score is included for each toxin. SNX-482(Omega-theraphotoxin-Hg1a) is highlighted in purple. Bars at the top of the alignment depict the percent identity at each position of the sequence. Conserved cysteines are highlighted in brown. (*) Identical residues (**b**) Surface representations of the Jingzhaotoxin-XI (left) and the SNX-482 (right) colored by residue type: positively charged (blue), negatively charged (red), and polar (white), hydrophobic (green). Both toxins’ structures are in the same orientation according to structural alignment. (**c**) The representative initial simulation system of the CG model (left) and the all-atom setup (right).

Template structures for SNX-482 were searched with BLAST [21], and highly similar sequences with known 3D structures were aligned (Figure 1a). Jingzhaotoxin-XI (PDB code: 2a2v) was selected as a template, and a comparative model of SNX-482 was generated with MODELLERv9.2 [22]. A total of three single structures from the NMR ensemble were selected and equilibrated by all-atom molecular dynamics (MD) simulations for 40 ns, each. The most representative structure of jingzhaotoxin-XI was obtained by a K-mean clustering (RMSD Ca-based with a 0.2 nm cutoff) analysis from the MD conformational ensemble employing the gromos++ software package [23] and then selected a template for the SNX-482 modeling. Comparative models (~100) were generated by satisfying spatial restraints via MODELLERv9.2. These models were grouped into three central clusters, whose centroids were subjected to 40 ns MD simulations. The final SNX-482 structural model corresponds to the centroid of the most representative cluster of the MD trajectories, as summarized in Supplementary Figure S1. A depiction of the final model, emphasizing distribution of positive charges, is shown in Figure 1b.

### 2.2. Coarse-Grained Molecular Dynamics Simulations

Coarse-Grained (CG) MD simulations were performed with the CUDA accelerated version of GROMACS 2021 [24], employing the Martini 3 force field [25]. All simulations were carried out in the NPT ensemble at 1 atm and 298 K in semi-anisotropic conditions, with pressure control via the Parrinello-Rahman barostat [26] and temperature control with the velocity-rescale thermostat. A 1.1 nm cutoff was employed for real-space electrostatics and Van der Waals (VdW) interactions with a smoothing function. Long-range Coulombic interactions were computed with the Particle-Mesh Ewald method (PME). A time-step of 20 fs was utilized for the MD integrator. The SNX-482 was converted to CG particle with Martinize [27]. An elastic-network constrained toxin configuration with lower and upper cutoffs of 0.5 nm and 0.9 nm, respectively. A force constant of 700 kJ/(mol nm^2^) was used.

Membrane bilayer patches were built via the CHARMM-GUI with the Martini Bilayer Maker module [28]. Mixtures of neutral (1-palmitoyl-2-oleoyl-sn-glycero-3-phosphocholine or POPC) and charged (palmitoyl-2-oleoyl-sn-glycero-3-phospho-rac-(1-glycerol) or POPG) at five different ratios were generated (1) POPC only, (2) POPC:POPG: 1:3, (3) POPC:POPG: 1:1, (4) POPC:POPG: 3:1 and (5) POPG only. In all cases, counter ions were added to neutralize the systems. The CG systems consisted of 109531 supraparticles with an average box size of 21 × 21 × 29 nm (see Figure 1c left). For each membrane composition, four independent simulations with the toxin placed at 12 nm from the outer leaflet of the membrane and in random orientations were carried out for 1 µs

### 2.3. All Atom Molecular Dynamics Simulations

All-atom (AT) MD simulations were carried out with the NAMDv2.13 CUDA accelerated simulation engine [29], the CHARMM36 parameter set for proteins, ions, and lipids, and the TIP3P water model [30]. All simulations were carried out at the NPT (1 atm and 298 K) ensemble with Langevin dynamics for temperature and semi-anisotropic piston fluctuation control. Long-range electrostatics were computed with the PME method with a relative tolerance of 1 × 10^−6^ and a cutoff of 1.2 nm for Van der Waal (VdW). Real-space electrostatic interactions and a switching function were applied between 1.0 and 1.2 nm. We used multiple time-step algorithms that consisted of 2 fs steps for bonded interactions and real space non-bonded interactions and 4 fs steps for the reciprocal space electrostatic interactions. All bonds involving hydrogens were constrained via the SHAKE algorithm [31].

To investigate membrane partitioning of the toxin, we generated five bilayer patches of ~11.5 × 11.5 nm containing 308 lipids with an axial length of 8.4 nm, employing the same compositions as for CG simulations. Counter ions were added to neutralize the systems. In these simulations, toxins were placed at ~2.5 nm from the outer leaflet of the membrane with different initial poses. The z-axes in all the simulation systems were set normal to the membrane plane. Overall, more than 30 μs of simulation time were performed in this study. A depiction of representative systems for the CG (left) and atomistic (right) systems are presented in Figure 1c.

### 2.4. Membrane Partitioning and Insertion Probability

Without previous knowledge of the docking pose of SNX-482 towards the membrane and its binding mode with different lipid compositions, we analyzed this process as follows. We monitored the distance along the z-axis between the toxin’s center of mass (COM) and the COM of the membrane. Once this distance reached a plateau, we computed the fraction of time each residue was inserted within the membrane. To this end, we took the COM of each phospholipid head from both sides of the membrane and fitted a straight line (z = ax + b). An insertion event was counted when the z coordinates of the COM of a residue fell below z, and the insertion probability of residues (Ip) is the number of insertion events that a particular residue undergoes divided by the number of frames in the trajectory. For each MD simulation, we defined an Insertion Score (IS) as follows:

(1) $$\mathrm{IS}\;=\;\sum_{i\;=\;1}^{\mathrm{NR}}\mathrm{Ip}$$

where NR is the total number of residues of SNX-482, the first insertion event to be computed was the one that occurred when the distance of the toxin COM and z was less than 0.5 nm. This requirement excludes spurious collisional contacts not related to a membrane-binding process.

### 2.5. Free-Energy and Potential Energy Calculations

Potential of Mean Force (PMF) calculations were carried out with the NAMD v2.14 software [29] employing well-tempered meta-dynamics [32,33] and the Colvar module of NAMD [34], in which a time-dependent potential (V_t+dt_) is applied on a collective variable (C_v_) with the following formulation:

(2) $$V_{t\;+\;\delta t}\left(C_{v}\right)\;=\;V_{t}\left(C_{v}\right)\;+\;W_{0}e^{-V_{t}\left(C_{v}\right)/k_{B}\Delta T}\prod_{i\;=\;1}^{N_{\mathrm{cv}}}G\left(C_{\mathrm{vi}};d_{i}\right)$$

N_cv_(C_v_) is the number of C_v_, and W_0_ and d_i_ are the height and bin width, respectively. G is a one-dimensional repulsive Gaussian “hill” with centers located at previously visited regions, as follows:

(3) $$G\left(C_{\mathrm{vi}};d_{i}\right)\;=\;\exp\left(-\frac{{\left(C_{\mathrm{vi}}\left(q;t\right)\;-\;C_{\mathrm{vi}}\left(q;t\prime \right)\right)}^{2}}{2d_{C_{\mathrm{vi}}}{}^{2}}\right)$$

By reweighting the height of each hill of Equation (2), a better convergence can be achieved. ΔT is the bias temperature. For long simulations, when V(C_v_) has converged, is a good approximation of the PMF:

(4) $$\mathrm{PMF}\left(C_{v}\right)≅\;-\frac{T+\Delta T}{\Delta T}V{\left(C_{v}\right)}_{t\to \infty }+C$$

T is the simulation temperature (298 K), and C is a constant to set the lowest PMF value to zero.

We started from the bound state identified in AT simulations for POPC:POPG 1.0 and 3:1, herein referred to as 1:0 and 3:1 systems. We projected the adsorption process into the distance along the z-axis between the protein and the membrane COM, and thus, Cv space was one-dimensional. Orientation, translation on the x-y plane, and conformation (ɑ-carbon RMSD) degrees of freedom of the toxin were restrained to the bound state configuration with harmonic potentials. The orientation matrix for the reference structure was defined via a quaternion formulation [35]. Membrane COM was also restrained. Harmonic constants, with the corresponding unit for each Cv, were 10 kcal/mol. The box sizes were extended along the z-axis to 130 Å.

Before production runs, the systems were relaxed for 50 ns in the bound states with the aforementioned restraints. Subsequently, well-tempered meta-dynamics were applied to the COM z-distance between the toxin and membrane and sampled between 13 Å and 45 Å. Soft harmonic walls were applied at these limits. The reaction coordinate was discretized every 0.5 Å (d_i_ of Equations (2) and (3)). We initially employed a hill weight of 1.0 Kcal/mol (W_0_ of Equation (2)) for both systems and a bias temperature of 1500 K. A Gaussian hill was added every 200 time-steps. However, for the 3:1 system, these parameters were not sufficient to enhance sampling along the reaction coordinate, even after 150 ns simulation. We increased the hill width to 3 Kcal/mol for the 3:1 system to improve sampling. The simulation times were 500 and 220 ns for the 3:1 and 1:0 systems, respectively.

Potential energy differences between the bound and unbound states were obtained from 4 sets of AT simulations: the bound and unbound states in the 1:0 and 3:1 systems. Same starting configurations from the PMF calculations were employed without any restraints for the bound condition. For the unbound state, the toxin was placed 4 nm away from the membrane and harmonically restrained along the z-axis to avoid toxin approximation to the membrane. All MDs were equilibrated for 50 ns, and production runs were carried out for 200 ns. Total potential energies (ΔE_TotPot_), VdW (ΔE_TotVdW_), electrostatic (ΔE_TotEle_), and bonded (ΔE_TotBond_) terms were obtained directly from the NAMD output files. These average energetic differences were further decomposed into pair-interactions terms involving toxin interactions (toxin-water, toxin-membrane, toxin-ions and toxin-toxin pairs) which were summed and decomposed into their VdW (ΔE_ToxVdW_) and electrostatic (ΔE_ToxEle_) terms. Interactions not directly involving the toxin (water–water, water–membrane, water–ions, membrane–membrane, membrane–ions, and ions–ions), were summed and decomposed into their VdW (ΔE_restVdW_) and electrostatic (ΔE_restEle_) terms. These calculations were performed over the trajectories sampled every 1 ps employing the pair-interaction module of NAMD. Errors were estimated by block averaging, extrapolating to infinite block size.

### 2.6. Diffusion Coefficient Calculation

The diffusion coefficient in 3D (water) and 2D (membrane) were calculated from mean squared displacements (MSDs) using the Einstein relation:

(5) $$D\;=\;\frac{1}{2d}\underset{t\to \infty }{\lim}\frac{\langle \left(r(t_{0}\;+\;t\right)\;-\;r\left(t_{0}\right){)}^{2}\rangle_{t_{0}}}{t}$$

(6) $$\langle \left(r(t_{0}\;+\;t\right)\;-\;r\left(t_{0}\right){)}^{2}\rangle_{t_{0}}\;=\;\mathrm{MSD}$$

where d corresponds to the number of dimensions (three and two for bulk and lateral diffusion, respectively); r is the (unwrapped) position vector of the center of mass (COM) at time t. Averaging was performed over multiple windows and times origins (t_0_). For bulk diffusion, a 1 μs MD in pure water was performed. Recent reports have described that finite-size effects when employing periodic boundary conditions and asymmetric box shapes (typical conditions for membrane simulations) arise in lateral diffusion coefficients within membranes in MD simulations [36]. Therefore, for lateral diffusion calculations, the periodic box of the last configuration from the MD with the highest IS was enlarged to a lateral size of 16 nm, and the simulation time was extended to 2 μs. From these diffusion constants (D_sim_), we extrapolated lateral diffusion constants to an infinite box size (D^∞^) employing a Bayesian method (https://diffusion.lobos.nih.gov/bayes.html, accessed on 13 December 2021)) that employs the Periodic Safmann-Delbrück model. D^∞^ extrapolation eliminated finite-size effects. We also computed lateral diffusion coefficients for the lipids (COM of polar heads). For more details on the specific parameters used for these calculations, please refer to Supplementary Tables S1 and S2.

## 3. Results and Discussion

The general aim of the present study was to determine if the negatively charged SNX-482 partitions into lipid membranes and investigate the importance of the anionic charge composition in this process. Many GMTx share a conserved hydrophobic patch surrounded by a ring of positively charged residues that have been proposed to promote peptide affinity for negatively charged lipid membranes [5,8,37]. Note that SNX-482 shares only five positively charged residues with the rest of the sequences aligned (Figure 1a) and that the comparative model of SNX-482 shows only three positive charges within the vicinity of the hydrophobic patch (Figure 1b). This raises the question about the ability of SNX-482 to interact with the membrane despite having an incomplete ring of positive charges and how negatively charged lipids may influence the binding of a peptide carrying a net negative charge.

### 3.1. SNX-482 Partitions into Partially Anionic Membranes in a Conserved Manner

To investigate in detail membrane partition, we carried out molecular dynamic simulation in CG and AT resolutions of SNX-482 in the presence of lipid bilayer with different ratios of neutral (POPC) and anionic lipids (POPG). Mixtures of POPG/POPC have been used for experimental measurements of GMTx/membrane partitioning [12,15]. Only neutralizing ions were added, keeping the ionic strength to a minimum and preventing surface charge screening from reducing the impact of membrane charges [15]. We considered two parameters to evaluate membrane insertion: (1) the fraction of simulation in which at least one insertion event was observed and (2) the insertion score detailed in the method section. Table 1 summarizes the results of four simulations in each condition, i.e., lipid composition, and for each resolution level, i.e., CG or AT. For CG simulations, we did not observe insertion events in any of the four simulations carried out with neutral membrane (POPC). Insertion events were observed in all other conditions. More notably, when considering only simulation with insertion events, IS values fall within the error margins for all anionic membranes, from POPC: POPG (3:1) to full POPG. This observation would lead us to conclude that anionic lipids are necessary for the toxin insertion process. However, only in one of the four AT MD simulations with a neutral membrane did we observe a successful membrane insertion. Thus, membrane binding, although disfavored, is still possible without anionic lipids.

We also observed a significant preference toward partially charged membranes in AT simulations, particularly for the 3:1 condition, and that fully charged membrane also disfavors membrane insertion. Overall, IS appears more sensitive to the membrane composition in AT simulation than CG. A partial explanation may be that COM values for each residue are closer to the protein backbone in CG, and thus, a fraction of the shallower insertion event counted in AT is missing in CG. In AT simulations, IS appears very sensitive to the ratio of neutral to anionic lipids, reaching an optimum with a 3:1 ratio, about three-fold higher than the other mixed membranes. In contrast, pure POPC or POPG seems equally unfavorable to membrane partition. A preference for a partially charged membrane over neutral ones has been observed experimentally with Protox II using plasmon resonance and all-atom simulation [14].

**Figure 2:**
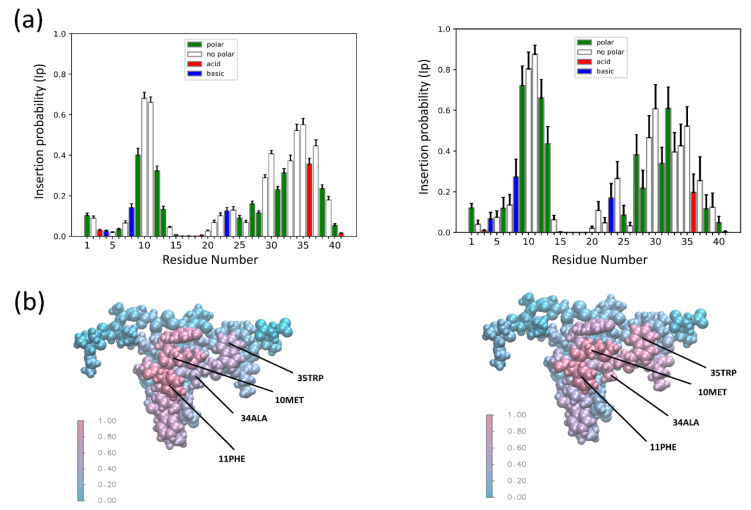
The global insertion probability (Ip) for coarse grain (CG) and all atom (AT) MD simulations. (**a**) The CG (left) and AT (right) insertion probability (Ip) of all MD simulations where there was successful insertion; n = 13 (CG) and n = 10 (AT). (**b**) VdW representations of SNX-482 colored by average Ip of all MD simulations where there was successful insertion. AT (left) and CG (right). Labels point to residues identified in all successful insertions.

**Figure 3:**
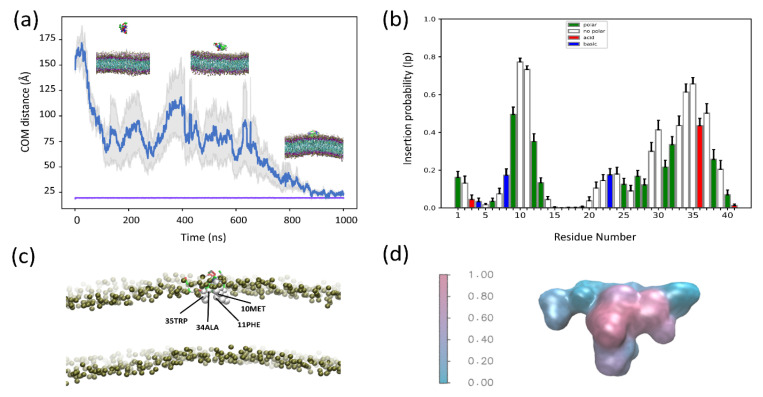
Coarse-grained MD simulations of SNX-482 and POPC: POPG (1:1) (**a**) Average distance as a function of time between the center of mass (COM) of SNX-482 and the (COM) of the membrane bilayer for four independent simulations. The gray colored region corresponds to the mean error of the distances. The purple line represents the position of the membrane outer leaflet. (**b**) Average insertion probabilities (Ip) for each residue. (**c**) Snapshot of membrane embedded toxin labelling residues with high ip (**d**) Surface representation of SNX-482 colored by Ip.

**Figure 4:**
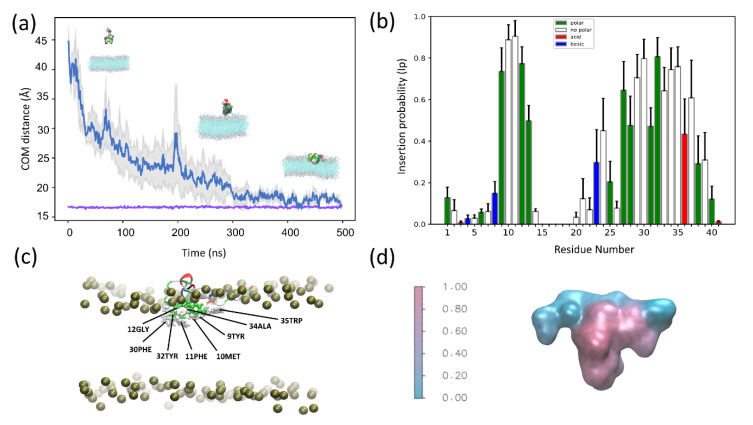
All-atom MD simulations of SNX-482 and POPC: POPG (3:1) (**a**) Average distance as a function of time between the center of mass (COM) of SNX-482 and the (COM) of the membrane bilayer for four independent simulations. The gray colored region corresponds to the mean error of the distances The purple line represents the position of the membrane outer leaflet. (**b**) Average insertion probabilities (Ip) for each residue. (**c**) Snapshot showing SNX-492 residues embedded in the membrane. (**d**) Surface representation of SNX-482 colored by Ip.

To explore the differences between the CG and AT descriptions in more detail, we computed the average Ip for each residue for all simulations in which there was a successful insertion event, regardless of the membrane composition (Figure 2a). Overlaying color-coded Ip values over a surface representation of SNX-482 allows us to visualize the global binding pose (Figure 2b). These results show that membrane partitioning residues are conserved regardless of membrane composition or resolution in all trajectories with at least one insertion event. We observe a bimodal distribution encompassing similar segments of the amino acid sequence in both cases. The first segment would span from residues 9 to 12 having an Ip > 0.2. Following a similar criterion, the second segment spans 29 to 39. The insertion process was investigated in more detail on simulations with the membrane composition that reported the most significant proportion of insertion events. These correspond to POPC: POPG ratios of 1:1 and 3:1 for CG and AT. The time evolution, average Ip for each residue, and binding pose are shown for these two cases: Figure 3 corresponds to CG simulation and Figure 4 to AT MD. Results for the other membrane compositions are reported in Supplementary Figures S2–S12. In both systems, the toxin comes into close contact with the membrane and reaches a plateau (below 0.5 nm) that remains stable for the final 200 ns of the simulation (Figure 3a and Figure 4a). Note that the system’s volume is larger for CG, and SNX-482 start 12.5 nm away from the membrane in CG in contrast to the 2.5 nm for AT. Consequently, it takes more than 800 ns for the toxin to contact the membrane in CG. During this time, SNX-482 is expected to explore all orientations before insertion, thus not being influenced by the initial pose. Nevertheless, we set different initial poses in each of the four CG simulations. In AT, the time to the first toxin-membrane contact was about 300 ns, more likely influenced by the initial pose. However, the gray area depicted in AT simulation trajectory suggests that even at such a short distance, the initial pose was not a relevant factor in the final configuration of the toxin-membrane complex.

To identify residues that contribute to membrane insertion, we computed the average Ip values of the final 150 ns of CG simulation with a 1:1 POPC: POPG membrane (Figure 3b). We chose the 3:1 POPC:POPG membrane for AT MD as it corresponds to the lipid ratio with the highest IS. In this case, we computed the average Ip for the final 200 ns (Figure 4b). In CG, Met10, Phe11, Phe30, Ala34, Trp35 dwell below the lipid head more than 50 % of the time (Figure 3c). This is in close agreement with AT MD, where Tyr9, Met10, Phe11, Leu29, Phe30, Tyr32, Ala34, Trp35 felt below the lipid head more than 60% of the time (Figure 4c). A surface representation of the toxin colored by average Ip values (Figure 3d and Figure 4d) shows that SNX-482 binding converges to the same pose with both CG and AT simulations. To evaluate the long-term stability of the AT binding pose, we took the last snapshot of one of the POPC: POPG 3:1 simulations and ran it for an additional 2 μs in an extended cubic box (see Section 2.5). IS values computed in 0.5 μs windows exhibit a monotonic increment and reach 21 in the last window while maintaining the binding pose (Supplementary Figure S14). Residues 23 to 39 kept increasing their Ip values over time (compare Figure 4 with Supplementary Figure S14). A rough linear estimation of the IS time-derivative (yields a value near 2 IS units per microsecond (Supplementary Figure S15). If residues 8 to 14 and 20 to 40 are the only membrane binders that, at equilibrium, reach an Ip value near one, IS maximum would be 26 (see Equation (1)). With an IS time-derivative of two, an additional 3 μs would be necessary to reach a final IS value and, thus, a stable binding pose. Such a slow stabilization of the membrane-toxin complex raises a cautionary note on information derived from short simulations.

The COM-based calculations may underestimate insertions and miss events where only the side chain falls below the membrane plane. Thus, we computed Ip profiles considering COM of the residues’ side chains (Figure S16) and revealed a slight reduction in Ip for polar and charged residues. IS calculated with this new criterion were virtually identical (Table S3). These restricted Ip measurements were also carried out for the last 0.5 µs of the extended simulation in the 3:1 system (Supplementary Figure S17A). We confirmed a reduction in Ip for polar residues, particularly from the first insertion patch encompassing residues 9 to 12. We also carried out Ip calculations employing a stricter criterion: a residue was considered inserted if it laid below C1 carbon of lipids’ fatty acid chain (Figure S17b). The latter is consistent with polar residues remaining closer to the polar heads while hydrophobic residues will go deeper in the membrane hydrophobic core.

We computed typical membrane descriptors for all compositions with the toxin unbound (first 50 ns simulation) to investigate if anionic phospholipids may influence membrane properties. Specifically, we measured area per lipid and membrane thickness (Table S2) and found no significant differences between the systems. Furthermore, as discussed below, toxin diffusivity is not affected by the presence of anionic lipids. We also explored whether changes in conformational stability might be relevant. We found that α-carbon RMSD for the bound configuration of the 1:0 and 3:1 systems exhibit similar fluctuations (Supplementary Figure S19a) and nearly identical inserted poses (Supplementary Figure S19b). We also corroborated that conformational freedom is significantly reduced upon membrane partitioning (Supplementary Figure S19c). Overall, the take-home message derived from this analysis is that both CG and AT identify a conserved binding mode towards the membrane surface with a preference over partially anionic composition.

### 3.2. The Role of Electrostatic Interactions in SNX 482 Membrane Partitioning

**Figure 5:**
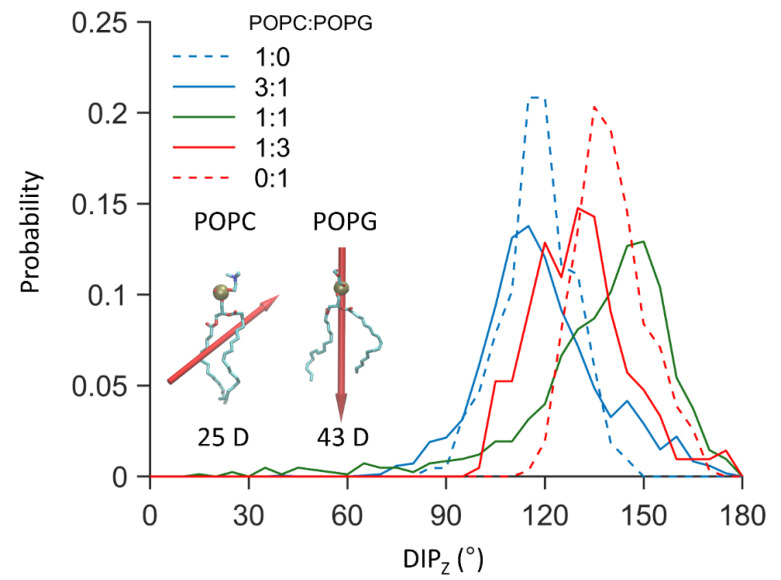
Probability distribution of the dipole angle with respect to the positive z-axis of membrane inserted SNX-482. Insert shows a licorice representation of POPC and POPG molecules with their respective dipole shown as red arrows, arrow size is proportional to the dipole magnitude of the phospholipid. The data was obtained from the unrestrained AT simulation showing spontaneous binding.

It is seemingly contradictory that electrostatic forces favor the binding of negatively charged SNX-482 to membranes containing anionic lipids. However, because the size of the toxin is relevant, the dipole moment is a more appropriate parameter to understand electrostatic forces. Indeed, as shown in Figure 5, the dipole moment of the bound toxin has a preferred orientation whose angle increases with the ratio of POPG, whose dipole orientation is perpendicular to the membrane plane and points to the interior. Thus, electrostatic forces would help align SNX-482 and POPG dipoles while the optimal orientation for membrane insertion falls around 100–120°.

**Figure 6:**
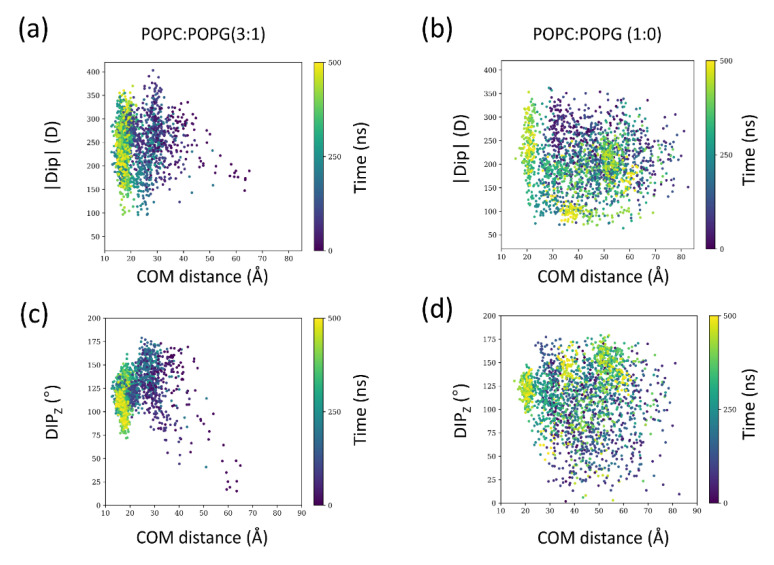
Dipolar interactions as a function of membrane distance. (**a**) dipole magnitude (|Dip|) and (**c**) dipole angle (Dip_z_) to the z-axis of SNX-482 for the 3:1 system. (**b**) dipole magnitude and (**d**) dipole angle with respect to the positive z-axis of SNX-482 for the 1:0 system. Points are colored according to the time in the simulation. In each condition, data from all four simulations were included. The data was obtained from the four unrestrained AT simulations displaying spontaneous binding simulations.

To get further insights into the dynamic behavior of the toxin electric dipole along the binding process, we evaluated how the toxin molecular dipole changes as the partitioning proceeds for the 3:1 and 1:0 systems (for all four AT simulations), corresponding to the ones with the highest and lowest number of binding events, respectively (Figure 6).

Compared to the 1:0 condition, in the 3:1 system, as the distance to the membrane decreases, allowed orientations (Dip_z_) are diminished (Figure 6c,f). Moreover, for the 3:1 system, the magnitude of the dipole is larger. Thus, dipole-dipole interactions appear relevant to orient and lock the toxin in a configuration that favors binding. We followed the same descriptors for the extended 2 μs simulations of the 3:1 system (Supplementary Figure S18). Quite interestingly, the orientation is maintained, but the dipole magnitude as time progresses is reduced and reaches even lower values than bulk conditions (see inset of Supplementary Figure S18A). The presence of anionic lipids appears to have a kinetic effect that facilitates the correct encounter between the membrane and SNX-482. This finding is in agreement with previous studies on a different family of peripheral membrane proteins, proving that the macrodipole of the peptide orientation plays a central role in membrane binding [35,36].

We estimated binding energies (ΔE; Table 2) to check whether the membrane composition also impacts the thermodynamics of membrane binding. To compute the total energetics of the partitioning, bound and unbound states of 3:1 and 1:0 systems were simulated for an additional 200 ns. We placed the toxin 4 nm above the outer membrane leaflet for unbound states and applied a soft-harmonic wall below this threshold only along the z-axis to avoid any approximation toward the membrane and minimize long-range interactions. We decomposed the total potential energy for the unbound and bound states into Van der Waals (VdW), electrostatic (Ele), and bonded (Bond) terms. We also decomposed these total energies for the pairs only involving the toxin (Tox) and the rest (Rest) of the interacting members (membrane, water, and counterions), which were also decomposed into its dispersion and electrostatic components. ΔE_TotPot_ values reveal that the 3:1 system exhibits favorable binding energy while the 1:0 system essentially shows no preference between the bound and free states; this is in line with the spontaneous binding results shown in Table 1.

Decomposing ΔE_TotPot_ into its ΔE_TotVdW_ and ΔE_TotEle_ components reveals that ΔE_TotVdW_ is deeper for the 3:1 system while ΔE_TotEle_ is positive for both, but four times higher for the 3:1 condition. A closer look at the decomposed interactions of the toxin and the rest of the system (ΔE_ToxVdW_, ΔE_ToxEle_, ΔE_RestVdW_ and ΔE_RestEle_) reveals that these basically cancel out for the 1:0 system. All interactions involving SNX-482 are not very different between the 1:0 and 3:1 systems. Consequently, the main difference between the binding processes for 3:1 and 1:0 ratios arises for the ΔE_RestEle_ and ΔE_RestVdW_ terms, with even a sign change for the latter. Therefore, membrane interactions with itself and water are differentially perturbed upon toxin binding onto the 3:1 and 1:0 systems. On the one hand, desolvating a charged membrane is more costly (thus the increase in ΔE_RestEle_), but in the same manner, the water removal and the deeper toxin insertion for the 3:1 condition increment dispersion interaction within the membrane. In the 3:1 system used there is also a larger concentration of ions, 30 to be exact. These are the counterions neutralizing anionic lipids. To check whether there was some preferential ion binding towards the membrane, we computed Na+ densities along the z-axis for the 3:1 system. We found that they concentrate in the membrane boundary with a small asymmetry between the upper and lower leaflet that can be attributed to the initial toxin placement closer to the upper leaflet (Supplementary Figure S20). Na^+^ density near the membrane increases in the toxin-bound system, indicating no counterion removal occurs upon membrane insertion. Comparing pair interactions between the toxin, ions, and water (Supplementary Table S5), the expected reduction in toxin–water electrostatic interactions upon membrane insertion is compensated by an increase in the interactions between the toxin and surface counterions. There is also a significant loss in VdW interactions with water compensated by the Vdw interactions with the membrane (see Table 2). Thus, surface counterions essentially cancel out the electrostatic cost of desolvating the toxin. We also observed a gain in interactions with surface ions upon binding for the 1:0 system but with only two ions, equivalent to 3.5 mM, it is insufficient to compensate the electrostatic cost of desolvation. Consequently, the toxin is more solvated when bound to neutral membrane. Thus, indirectly, anionic membrane composition by concentrating cations near the membrane surface reduces the cost of toxin desolvation necessary for binding.

**Figure 7:**
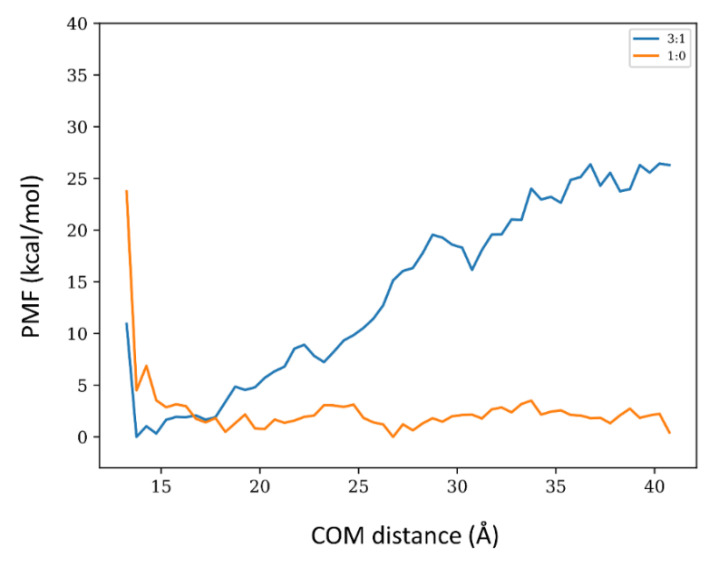
Exploratory Potential of Mean Force (PMF) of the axial transfer of SNX-482 bound to the membrane. To improve convergence, the toxin was orientationally, conformationally, and translationally (x-y plane) restrained with respect to the bound configuration and sampled up to a 4.2 nm distance from the membrane upper leaflet. In blue and orange, the 3:1 and 1:0 systems.

We carried out an exploratory potential of mean force (PMF) calculations for the 1:0 and 3:1 systems via well-tempered meta-dynamics (wt-MetaDym) to complement the energy calculations. To avoid noise and optimize these demanding calculations, translational (along with the x-y plane) orientational and conformational degrees of freedom were restrained to the bound state. With these restraints, PMFs were calculated as a function of the COM distances between the toxin and the membrane along the z-axis for both systems. Distances up to 4.2 nm were considered (Figure 7). At this distance, both systems do not exhibit detectable dipole alignment and magnitudes in solution (Figure 6 and Supplementary Figure S16). Since the adsorbed states exhibit a conserved binding mode regardless of the membrane composition (see Figure 2, Figure 3 and Figure 4), the main difference in adsorption strength arises from the cost of transferring the restrained toxin from the bound state to the bulk and, therefore, the entropic penalties associated with introducing and removing these restraints (and the standard state correction) should cancel out when determining relative adsorption affinities. For a convergence analysis of the wt-MetaDym calculations, please refer to Supplementary Figure S20. The energetic calculations of the PMFs shown in Figure 7 clearly indicate that the 3:1 membrane composition favors toxin binding, while the 1:0 shows essentially a rather flat free-energy profile for the restrained axial transfer. A thermodynamic cycle in which we add the free energy cost of restraining the bound state system will be positive but small, due to low conformational and orientational freedom of the bound toxin while in the unbound state, the difference in free energy will be negative and larger for the more extensive conformational and orientational freedom of the toxin in solution reducing the absolute binding free-energy differences. We did not carry out these calculations as we were mainly interested in the relative difference between both conditions. Considering the conserved binding poses, it is reasonable to assume that these terms will cancel out. According to the energy calculations shown in Table 2, the potential energy difference of absorption for the 1:0 system is essentially zero. It is expected that the gain in entropy in the unbound state, which can be roughly approximated by removal the aforementioned restraints, will further reduce the affinity of the toxin towards the 1:0 system. We did not carry out these calculations as we were mainly interested in the relative difference between both conditions. Considering the conserved binding poses, it is reasonable to assume that these terms will cancel out. According to the energy calculations shown in Table 2, the potential energy difference of absorption for the 1:0 system is essentially zero. It is expected that the gain in entropy in the unbound state, which can be roughly approximated by the restraints removal, will further reduce the affinity of the toxin towards the 1:0 system.

From the measured partition coefficient for other toxins, the free energy change of membrane binding is in the order of −7 to −8 Kcal/mol [12]. Our PMF calculations for the 3:1 system exhibit a (partial) binding free energy in the order of −20 kcal/mol (Figure 7). However, this value is expected to increase when removing the energetic penalty of the employed restraints, in particular the conformational and orientational ones imposed over the toxin, given that there is a relevant change in the dipole magnitude and orientation between the unbound and bound states (see Figure 6 and Supplementary Figure S18). Furthermore, the standard state correction (logarithm of the ratio between the sampled restrained volume and the standard volume) leads to a further increment of around 3 Kcal/mol. The discrepancy with ΔE_TotPot_ (see Table 2) can be attributed to a relevant entropic loss expected from the loss in translational, rotational, and configurational degrees of freedom of the toxin upon binding, which is not compensated by interfacial water molecules. The precise nature of the membrane-toxin interaction for the 3:1 condition is reflected by the ΔE_ToxVdW_ and ΔE_ToxEle_ shown in Table 2. Both terms are favorable, reflecting the polar, charged, and hydrophobic nature of the protein insertion within the membrane.

As a whole, these calculations strongly suggest that the partition process is energetically driven, and the anionic membrane composition, specifically the dipole moment, aids the optimal orientation and configuration for membrane insertion.

### 3.3. Toxin Diffusivity Is Reduced upon Membrane Insertion

To obtain insights on the diffusional effects of membrane partitioning, we computed the diffusion coefficient of the toxin in the aqueous phase (D_AP_) and the lateral diffusion coefficients (D_M_) when bound to the membrane for the AT POPC: POPG 3:1 system (see Section 2.5 for more details). Due to finite-size effects, we also extrapolated D_M_ values to macroscopic scales, D^∞^ [36,38]. These results are presented in Table 3, Table S1 and Table S2. Bulk diffusion for SNX-482 is one or more than one order of magnitude larger than lateral diffusion within the membrane for the microscopic (D^sim^) or macroscopic (D^∞^) estimations, respectively. The latter is expected due to the toxin-membrane interactions and the higher viscosity of the membranous phase. Indeed, a comparison between D_M_ values of SNX-482 and the lipids not regarding their composition reveals that the toxin diffusivity is determined mainly by the membrane, particularly for D^∞^ estimations (second column of Table 3).

**Figure 8:**
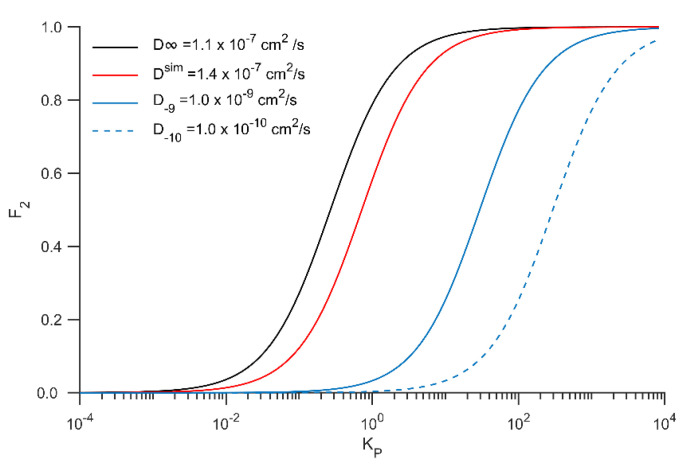
Fraction of binding from membrane bound toxin with respect to the total binding rate (F_2_) as function of the partition coefficient (K_p_) for different diffusion coefficients along the membrane. Calculations were carried out with equation 4 assuming R_a_ = 0.5 nm and SA:V = 1 × 10^6^ cm^−1^.

The question arises then on the impact of slower membrane diffusion on the preferred path to binding, i.e., directly from the bulk or through lateral diffusion along the membrane. Is RD sufficient to sustain a higher encounter rate for the membrane-bound ligand despite a near ten-fold slower diffusion? The seemingly simpler scenario of a diffusion-limited process in which all toxin-receptor encounters led to binding is not trivial and is the subject of intense debate in the literature, as for 2D (and 1D as well). The main caveat is that binding rates contain time and concentration dependencies [5,10,36,37]. Fortunately, the more realistic scenario in which toxin-channel binding is a “reaction-limited” process. Axelrod and Wang developed a simple RD theory for reaction-limited receptors either for reversible or irreversible binders [10]. In detail, it assumes the following: (i) the 2D and 3D processes are indirectly coupled by reversible adsorption on non-occupied regions of the surface; (ii) the receptor binding site is equally available from the bulk or from the surface and randomly distributed; (iii) the binding probability per collisions is low; (iv) receptor single-occupancy, (vi) a spherical geometry and (vii) equilibrium is reached, so the concentrations of ligand and receptor remain constant. With all these conditions satisfied and for the reversible ligand-receptor binding case, the fraction of its rate from the surface with respect to the total binding rate (F_2_) is:

(7) $$F_{2}\;=\;{\left[\left(\frac{3\Pi }{16}\right)\left(\frac{\sigma_{2}}{\sigma_{3}}\right)\left(\frac{\chi_{3}}{\chi_{2}}\right)\left(\frac{D_{3}}{D_{2}}\right)\left(\frac{R_{a}\;\mathrm{SA}:V}{K_{P}}\right)\;+\;1\right]}^{-1}$$

where *σ* and χ are the Brownian persistence distance (free path between collisions) and the fraction of collisions that lead to binding. For simplicity, we assume that these parameters are the same in bulk (σ_3_ and χ_3_) and membrane-bound (σ_2_ and χ_2_). R_a_ is the capture radius, which is in the order of nanometers. D_2_ and D_3_ are the toxin’s diffusion constants membrane-bound or free in solution, respectively. K_P_ is the partition coefficient between the membrane and bulk phases. Axelrod and Wang’s [10] original formulation was expressed in terms of absorption constant (K_ads_), being the quotient between surface (mol/cm^2^) and bulk (mol/cm^3^) concentration, it has units of cm^−1^. Assuming that all toxin remains on the surface in the membrane phase, K_p_ = (SA:V)K_ads_ being SA:V the surface area to volume ratio, a parameter that strongly depends on size and geometry. At the microscale and in perfect spherical geometry, SA:V is in the order of 10^6^ cm^−1^. Inspection of Equation (4) shows that F_2_ increases with K_P_ and D_2_. Figure 8 illustrates how these two variables influence F_2_. For the diffusion constants obtained in this work (D^∞^ and D^sim^), the RD mechanism is clearly the dominant process even for K_P_ near unity. We also estimated F_2_ for diffusion constants of 10^−9^ and 10^−10^ cm^2^/s that displace the curves to the right. In such cases, F_2_ approaches one when K_P_ > 1000. Some final points are worth mentioning: two parameters were defined as equal in the bulk and within the membrane; σ depends on the viscosity of the medium and should be more prominent within the membrane, thus reducing F_2_; χ may also increase in 2D given the reduced sampling space for random collisions, thus augmenting F_2_. Notwithstanding, it is reasonable to assume that apart from partially canceling each other in Equation (7), these factors will likely not differ by more than an order of magnitude with respect to bulk conditions. Finally, this model assumes that binding occurs with a low probability per collision as when a kinetic barrier for binding exists, as it is often the case in protein-ligand binding phenomena.

## 4. Conclusions

In this study and up to our knowledge, for the very first time, the membrane partitioning of the gating-modifier toxin SNX-482 was systematically studied via coarse-grained and all-atom molecular dynamics simulations. We determined that the toxin spontaneously binds to membranes in a conserved manner with a preference toward partially anionic membrane composition. The inserted protein patch contains a mixture of polar and hydrophobic residues. Energetic calculations strongly suggest that electrostatic interactions guide the partitioning process by orienting and locking the toxin in a state that enhances adsorption and that the protein–membrane interactions are stabilized by favorable Van der Waals interactions upon insertion. Lateral diffusion estimations revealed that the toxin mobility within the membrane is at least an order of magnitude slower when compared to bulk conditions. For partition coefficients above one, the RD mechanism is still the dominant with this reduction in diffusivity, according to the Axelrod and Wang model [10]. With large Kp, as for membrane containing anionic lipid, all binding is expected to occur via RD. Thus, a key factor determining the toxin binding mechanism is the partition of the toxin within the membrane.

## Figures and Tables

**Table 1:** Insertion Score (IS) of SNX-482 in lipid bilayers of different POPC/POPG ratio.

Lipid Composition	Coarse Grain		All-Atom	
	IS	Bindings/Run	IS	Bindings/Run
POPC	0	0/4	2.7 ± 2.5	1/4
POPC:POPG (3:1)	5.4 ± 2.3	4/4	13.4 ± 2.2	4/4
POPC:POPG (1:1)	7.9 ± 1.1	4/4	3.9 ± 2.3	2/4
POPC:POPG (1:3)	4.7 ± 3.1	2/4	5.5 ± 2.2	2/4
POPG	6.8 ± 2.3	3/4	1.3 ± 0.6	1/4

IS: insertion score, Bindings/Run: the ratio of simulations with at least one insertion event occurring while the membrane-toxin distance was less than 0.5 nm. The maximum possible IS values correspond to 41, when all residues are inserted in all frames.

**Table 2:** Average energy differences in kcal/mol between bound and unbound states for the 3:1 and 1:0 systems *.

Component	3:1	1:0
ΔE_TotPot_	−83.8 ± 7.4	5.5 ± 6.3
ΔE_TotVdW_	−122.8 ± 4.8	−4.0 ± 3.0
ΔE_TotEle_	36.7± 5.0	8.2 ± 4.8
ΔE_TotBond_	1.8 ± 2.6	1.4 ± 2.7
ΔE_ToxVdW_	−65.3 ± 3.2	−52.7 ± 2.8
ΔE_RestVdW_	−56.6 ± 4.0	48.7 ± 4.8
ΔE_ToxEle_	−76.0 ± 3.3	−53.8 ± 3.9
ΔE_RestEle_	111.4 ± 4.4	62.1 ± 4.6

* ΔE: Average energy differences between bound and unbound states in kcal/mol. TotPot: total potential energies); TotVdW: total Van der Waals energy; TotEle: total electrostatic energy; TotBond: total bonded energy; ToxVdW: Sum of pair-interaction Van der Waals energy terms involving SNX-482; ToxEle: Sum of pair-interaction electrostatic energy terms involving SNX-482; RestVdW: Sum of pair-interaction Van der Waals energy terms not involving SNX-482; RestEle: Sum of pair-interaction electrostatic energy terms not involving SNX-482.

**Table 3:** Diffusion coefficients for unbound and bound states.

Diffusion Coefficient	D^∞^ (cm^2^/s)	D^sim^ (cm^2^/s)
SNX-482 partitioned in POPC:POPG (3:1) (DM) ^1^	1.1 × 10^−7^	4.1 × 10^−8^
SNX-482 in solution (D_AP_)	--	1.0 × 10^−6^
POPC ^2^	1.0 × 10^−7^	1.5 × 10^−7^
POPC:POPG (3:1) ^1^	1.0 × 10^−7^	1.6 × 10^−7^
POPC:POPG (1:1) ^2^	1.0 × 10^−7^	1.3 × 10^−7^
POPC:POPG (1:3) ^2^	1.0 × 10^−7^	1.5 × 10^−7^
POPG^2^	1.0 × 10^−7^	1.4 × 10^−7^

D^∞^ is the diffusion coefficient extrapolated to an infinite size system. D^sim^ is the diffusion coefficient obtained from the AT MD simulations. ^1^ Diffusion coefficient in the membrane (DM) was calculated from a 2 μs AT MD in a 16 nm cubic box. ^2^ Diffusion coefficients calculated from 500 ns AT MD simulations.

## Data Availability

The data presented in this study are available upon request to the corresponding authors.

## Supplementary Materials

The following are available online at https://www.mdpi.com/article/10.3390/membranes12060595/s1, Tables S1–S5. Figures S1–S21.
