# Supplementary material for: Spider Toxin SNX-482 Gating Modifier Spontaneously Partitions in the Membrane Guided by Electrostatic Interactions
Source: Membranes (Basel). 2022 Jun 6;12(6):595. doi: 10.3390/membranes12060595 (PMC9231009; doi:10.3390/membranes12060595)
Supplement: Supplementary file 1 [file membranes-12-00595-s001.zip › membranes-1712456-supplementary.pdf]

Table S1. Extrapolation simulated diffusion constants (D^{sim}) to the infinite box size (D^{∞}) for different membrane compositions and different parameters for the Periodic Safmann-Delbrück model.

Lipids	Water viscosity ηf (Poise)	Membrane viscosity ηm (Poise cm)	Interleaflet coupling b (Poise/cm)	D^{sim} (cm^2/s)	D^{∞} (cm2/s)[1]
POPC	0.00311	0.3x10^{-7}	0.1 x 10^{7}	1.5 x10^{-7}	5.1 x 10^{-7}
	**0.00311**	**2.3 x10^{-7}**	**1.0 x 10^{7}**		**1.0 x 10^{-7}**
	0.00311	4.3 x10^{-7}	10 x 10^{7}		0.6 x 10^{-7}
	0.0089	0.3x10^{-7}	0.1 x 10^{7}		4.0 x 10^{-7}
	0.0089	2.3 x10^{-7}	1.0 x 10^{7}		0.9 x 10^{-7}
	0.0089	4.3 x10^{-7}	10 x 10^{7}		0.5 x 10^{-7}
POPC:POPG (3:1)	0.00311	0.3x10^{-7}	0.1 x 10^{7}	1.6 x10^{-7}	5.2 x 10^{-7}
	**0.00311**	**2.3 x10^{-7}**	**1.0 x 10^{7}**		**1.0 x10^{-7}**
	0.00311	4.3 x10^{-7}	10 x 10^{7}		0.6 x 10^{-7}
	0.0089	0.3x10^{-7}	0.1 x 10^{7}		4.0 x 10^{-7}
	0.0089	2.3 x10^{-7}	1.0 x 10^{7}		0.9 x 10^{-7}
	0.0089	4.3 x10^{-7}	10 x 10^{7}		0.5 x 10^{-7}
POPC:POPG (1:1)	0.00311	0.3x10^{-7}	0.1 x 10^{7}	1.3x10^{-7}	5.0 x 10^{-7}
	**0.00311**	**2.3 x10^{-7}**	**1.0 x 10^{7}**		**1.0 x 10^{-7}**
	0.00311	4.3 x10^{-7}	10 x 10^{7}		0.6 x 10^{-7}
	0.0089	0.3x10^{-7}	0.1 x 10^{7}		3.9 x 10^{-7}
	0.0089	2.3 x10^{-7}	1.0 x 10^{7}		0.9 x 10^{-7}
	0.0089	4.3 x10^{-7}	10 x 10^{7}		0.5 x 10^{-7}
POPC:POPG (1:3)	0.00311	0.3x10^{-7}	0.1 x 10^{7}	1.5 x 10^{-7}	5.1 x 10^{-7}
	**0.00311**	**2.3 x10^{-7}**	**1.0 x 10^{7}**		**1.0 x 10^{-7}**
	0.00311	4.3 x10^{-7}	10 x 10^{7}		0.6 x 10^{-7}
	0.0089	0.3x10^{-7}	0.1 x 10^{7}		4.0 x 10^{-7}
	0.0089	2.3 x10^{-7}	1.0 x 10^{7}		0.9 x 10^{-7}
	0.0089	4.3 x10^{-7}	10 x 10^{7}		0.5 x 10^{-7}
POPG	0.00311	0.3x10^{-7}	0.1 x 10^{7}	1.4 x 10^{-7}	5.1 x 10^{-7}
	**0.00311**	**2.3 x10^{-7}**	**1.0 x 10^{7}**		**1.0 x 10^{-7}**
	0.00311	4.3 x10^{-7}	10 x 10^{7}		0.6 x 10^{-7}
	0.0089	0.3x10^{-7}	0.1 x 10^{7}		4.0 x 10^{-7}
	0.0089	2.3 x10^{-7}	1.0 x 10^{7}		0.9 x 10^{-7}
	0.0089	4.3 x10^{-7}	10 x 10^{7}		0.5 x 10^{-7}

Parameters used in the extrapolation a Water height * H (nm) = 4, System lateral size L (nm) = 12 and Hydrodynamic radius R (nm) = 0.47 [1].

In bold, parameters an D^{∞} reported in Table 3.

[1]. Venable, R.M.; Ingólfsson, H.I.; Lerner, M.G.; Perrin, B.S.; Camley, B.A.; Marrink, S.J.; Brown, F.L.H.; Pastor, R.W. Lipid and Peptide Diffusion in Bilayers: The Saffman–Delbrück Model and Periodic Boundary Conditions. *J. Phys. Chem. B* **2017**, *121*, 3443–3457, doi:10.1021/acs.jpcb.6b09111.

Table S2. Extrapolation of simulated diffusion constants (D^{sim}) to the infinite box size (D^{∞}) for partitioned SNX-482 within POPC:POPG (3:1) and different parameters for the Periodic Safmann-Delbrück model.

toxin-membrane	Water height (nm)	System lateral size (nm)	Water viscosity ηf (Poise)	Membrane viscosity ηm (Poise cm)	Interleaflet coupling b (Poise/cm)	Hydrodynamic radius R (nm)	D^{sim} (cm2/s)	D^{∞} (cm2/s)
SNX-482 POPC:POPG (3:1)	6	16	0.00311	0.3×10^{-7}	0.1×10^{7}	1.08	4.1×10^{-8}	2.96×10^{-7}
			0.00311	2.3×10^{-7}	1.0×10^{7}			1.06×10^{-7}
			0.00311	4.3×10^{-7}	10×10^{7}			0.66×10^{-7}
			0.0089	0.3×10^{-7}	0.1×10^{7}			**1.87×10^{-7}**
			0.0089	2.3×10^{-7}	1.0×10^{7}			0.89×10^{-7}
			0.0089	4.3×10^{-7}	10×10^{7}			0.56×10^{-7}

In bold, parameters an D^{∞} reported in Table 3.

1. Venable, R.M.; Ingólfsson, H.I.; Lerner, M.G.; Perrin, B.S.; Camley, B.A.; Marrink, S.J.; Brown, F.L.H.; Pastor, R.W. Lipid and Peptide Diffusion in Bilayers: The Saffman–Delbrück Model and Periodic Boundary Conditions. *J. Phys. Chem. B* **2017**, *121*, 3443–3457, doi:10.1021/acs.jpcb.6b09111.

Table S3. Insertion Score (IS) of SNX-482 in lipid bilayers of different POPC/POPG ratios using the sidechain insertion criteria.

Lipid composition	All-atom	
	IS	Bindings/Run
POPC	2.5 ± 2.4	1/4
POPC:POPG (3:1)	13.3 ± 2.2	4/4
POPC:POPG (1:1)	3.8 ± 2.3	2/4
POPC:POPG (1:3)	4.2 ± 2.9	2/4
POPG	0.93 ± 0.6	1/4

IS: insertion score, Bindings/Run: the ratio of simulations with at least one insertion event occurring while the membrane-toxin distance was less than 0.5 nm and sidechain residues are above of lipids heads.

Table S4. Membrane properties for all membrane compositions during the first 50ns of simulations

Lipid composition	All-atom	
	Area per lipid (Å^2)	Membrane thickness (Å)
POPC	68.8± 0.1	37.3± 0.0
POPC:POPG (3:1)	68.5±1.8	37.5± 0.6
POPC:POPG (1:1)	68.8± 0.1	36.7± 0.0
POPC:POPG (1:3)	66.7± 0.2	37.5± 0.1
POPG	66.2±0.2	37.7± 0.1

Table S5. Average energy differences in kcal/mol between bound and unbound states for ion and water interactions with SNX-482 for the 3:1 and 1:0 systems.

Component	Coulomb	VdW
$\Delta E(3:1)_{toxin\text{-}wat}$	436.2 ± 18.1	89.2 ± 2.0
$\Delta E(3:1)_{toxin\text{-}ion}$	-459.8 ± 27.9	6.4 ± 0.5
$\Delta E(1:0)_{toxin\text{-}wat}$	71.5 ± 21.1	66.2 ± 1.9
$\Delta E(1:0)_{toxin\text{-}ion}$	-11.3 ± 8.6	0.1 ± 0.1

***ΔE**: Average energy differences between bound and unbound states in kcal/mol; toxin-wat and toxin-ion refer to the interaction energy between the SNX-482-ion and SNX-482-water pairs, respectively.

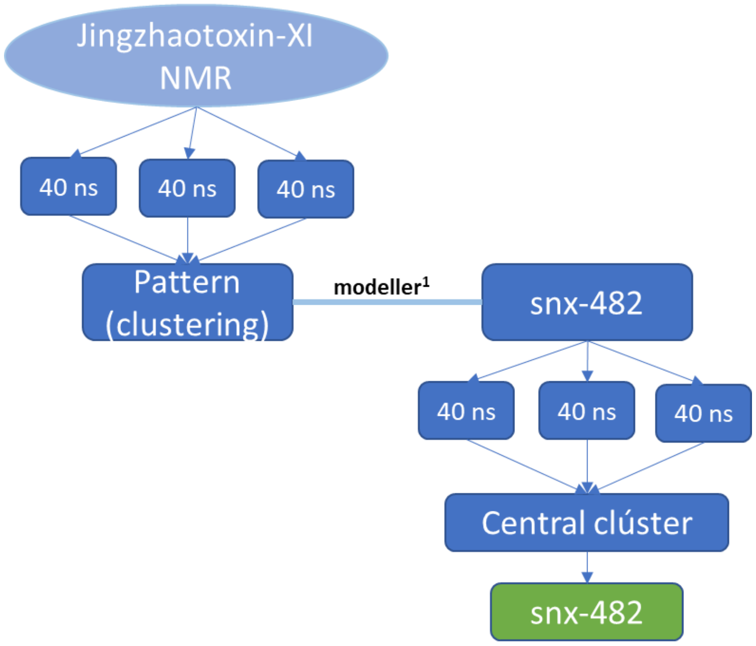

Figure S1. Comparative modeling scheme SNX-482. We randomly selected 3 PDB from Jingzhaotoxin-XI (Id: 2A2V) to perform independent 40 ns all-atom MD simulations. Afterward, we carried out comparative modeling with the central cluster of the simulations to produce 100 comparative models of SNX-482. We randomly selected 3 SNX-482 models and performed 40 ns all-atom MD simulations. The final SNX-482 comparative model corresponds to the central cluster of these simulations.

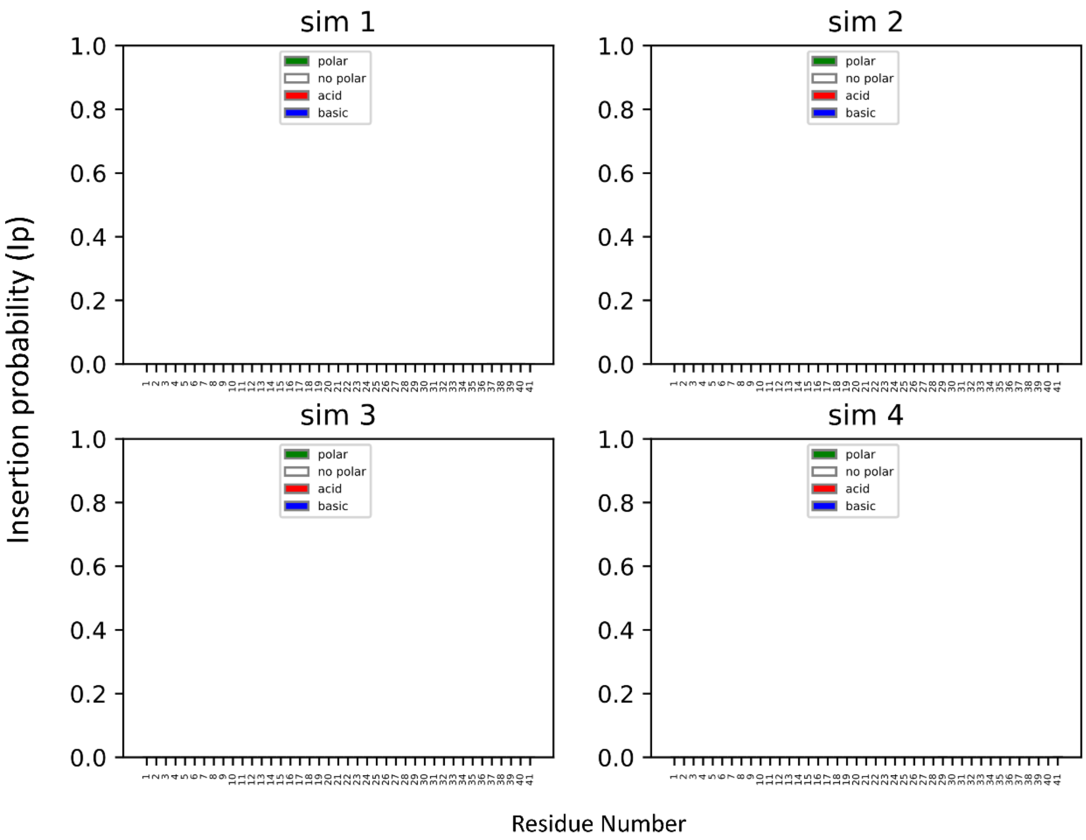

Figure S2. Insertions probability for each CG simulation of SNX-482 in pure POPC

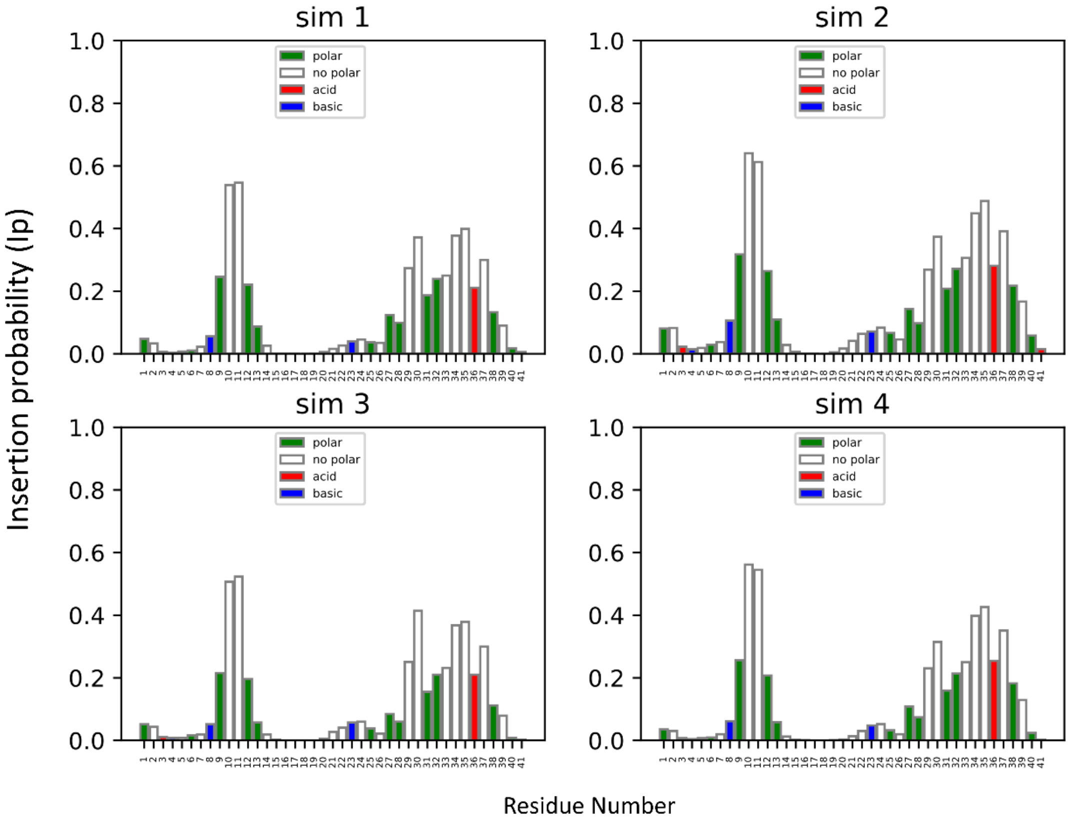

Figure S3. Insertions probability for each CG simulation of SNX-482 in POPC: POPG (3:1).

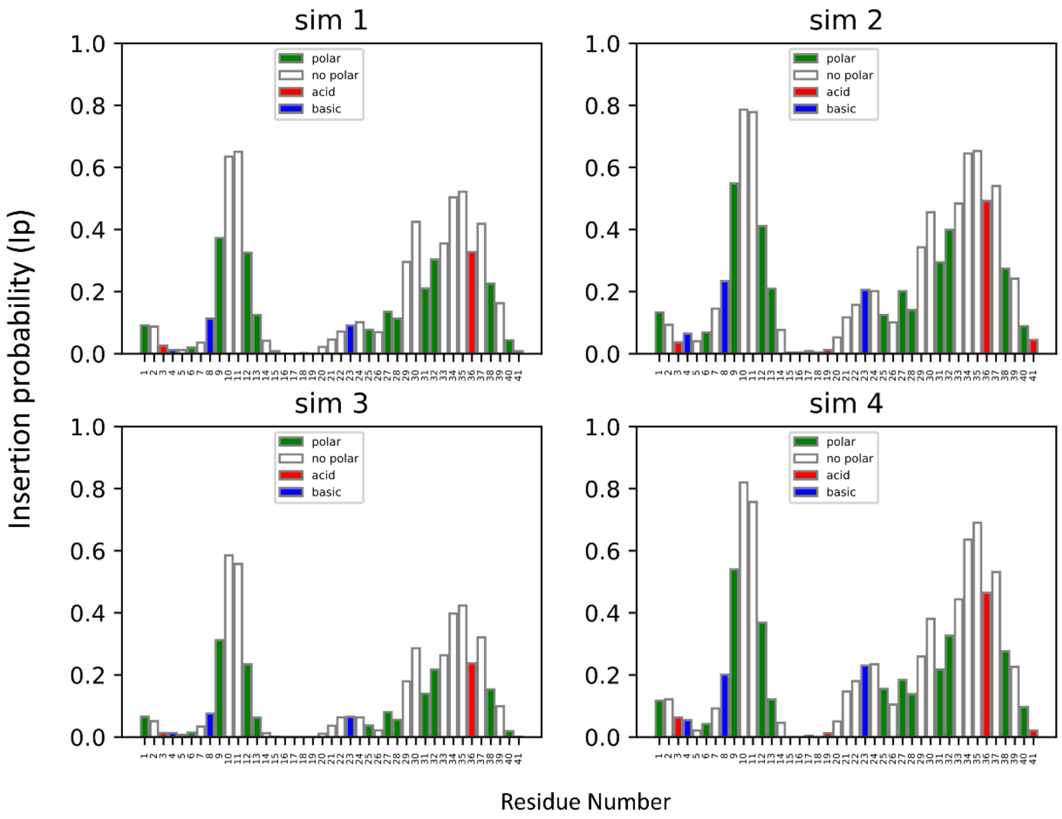

Figure S4. Insertions probability for each CG simulation of SNX-482 in POPC: POPG (1:1).

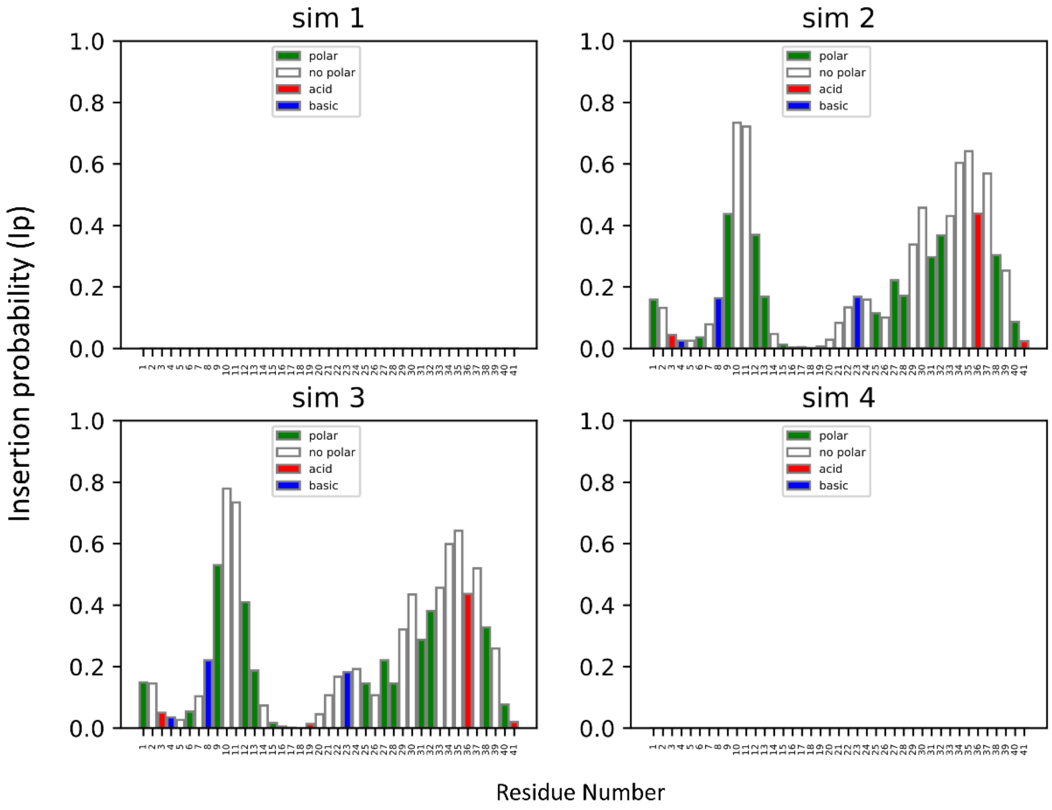

Figure S5. Insertions probability for each CG simulation of SNX-482 in POPC: POPG (1:3).

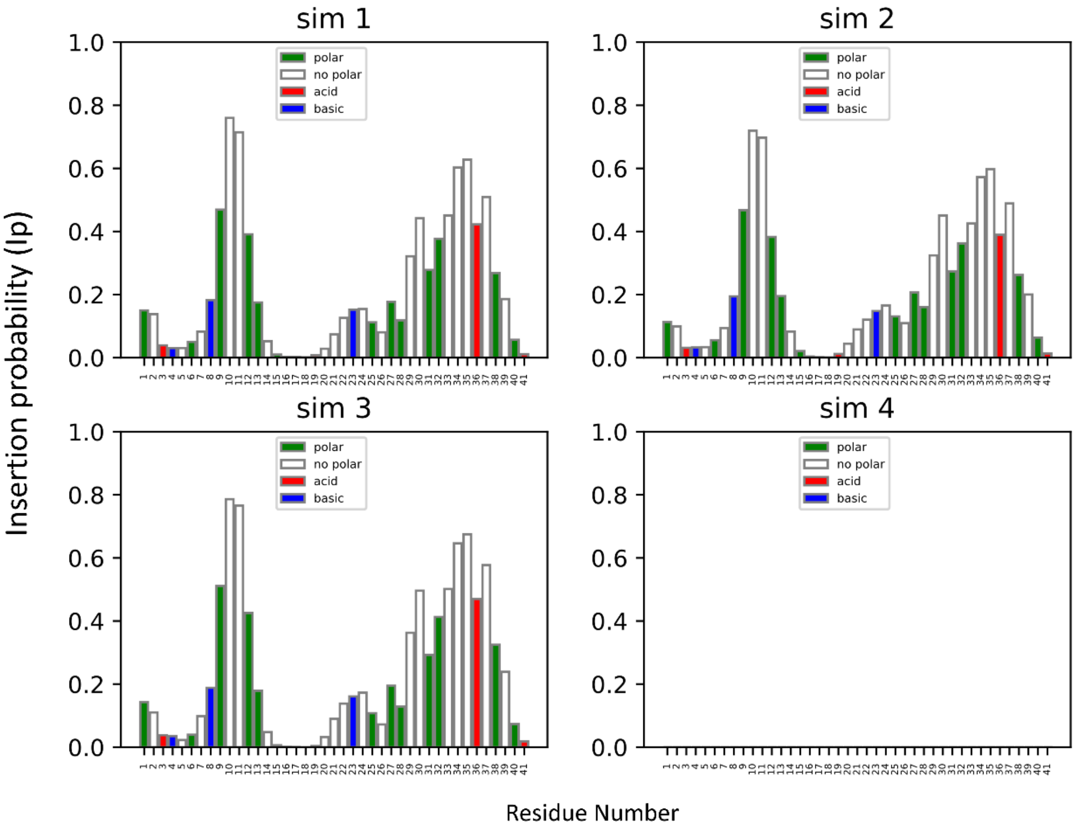

Figure S6. Insertions probability for each CG simulation of SNX-482 in pure POPG

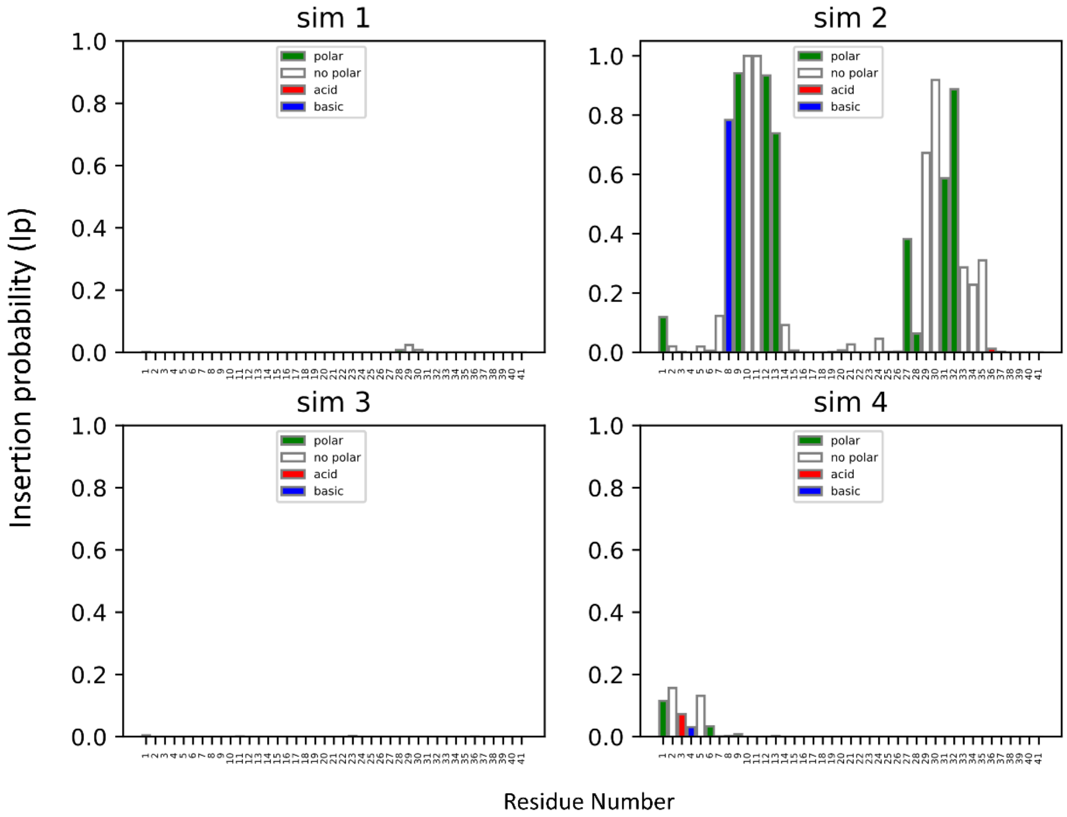

Figure S7. Insertions probability for each all-atom simulation of SNX-482 in pure POPC.

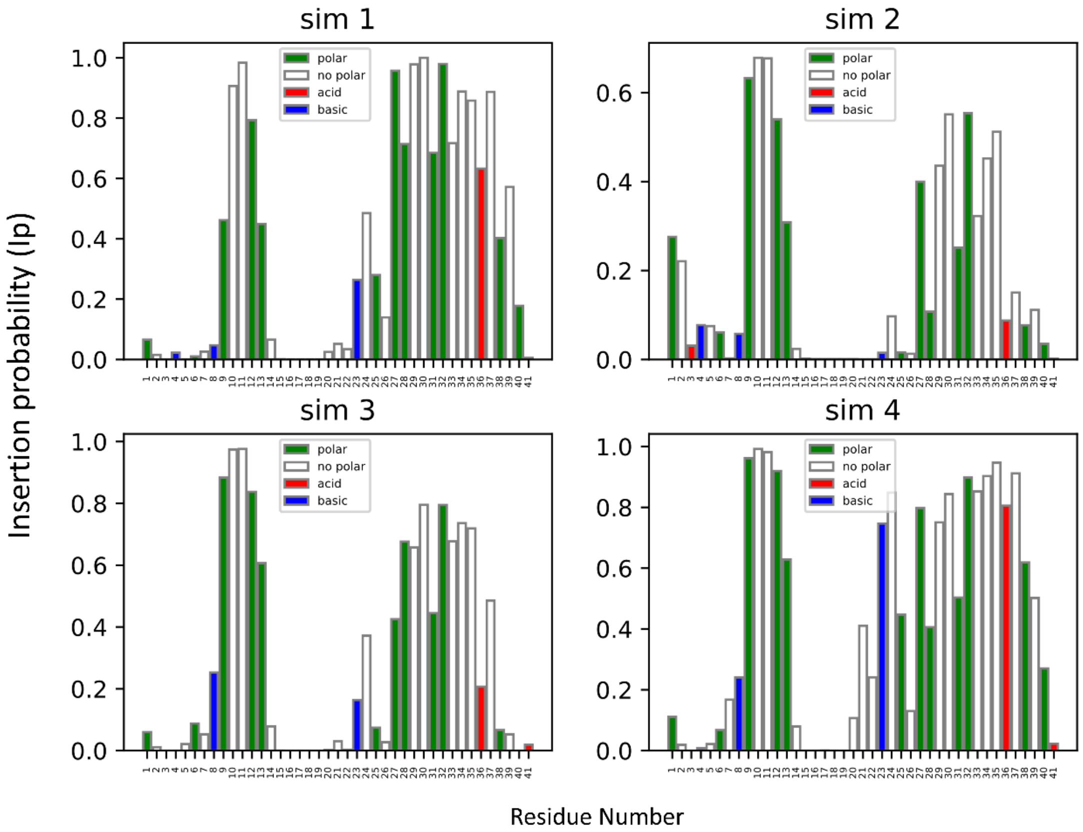

Figure S8. Insertions probability for each all-atom simulation of SNX-482 in POPC: POPG (3:1).

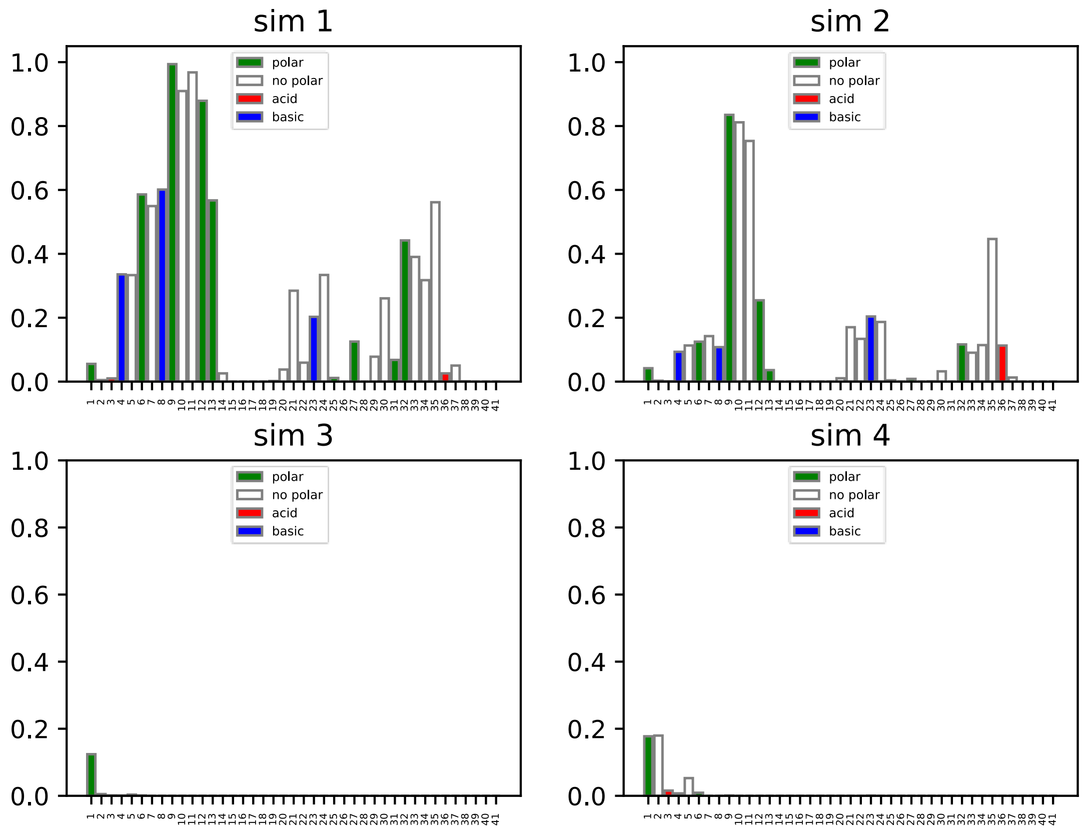

Figure S9. Insertions probability for each all-atom simulation of SNX-482 in POPC: POPG (1:1)

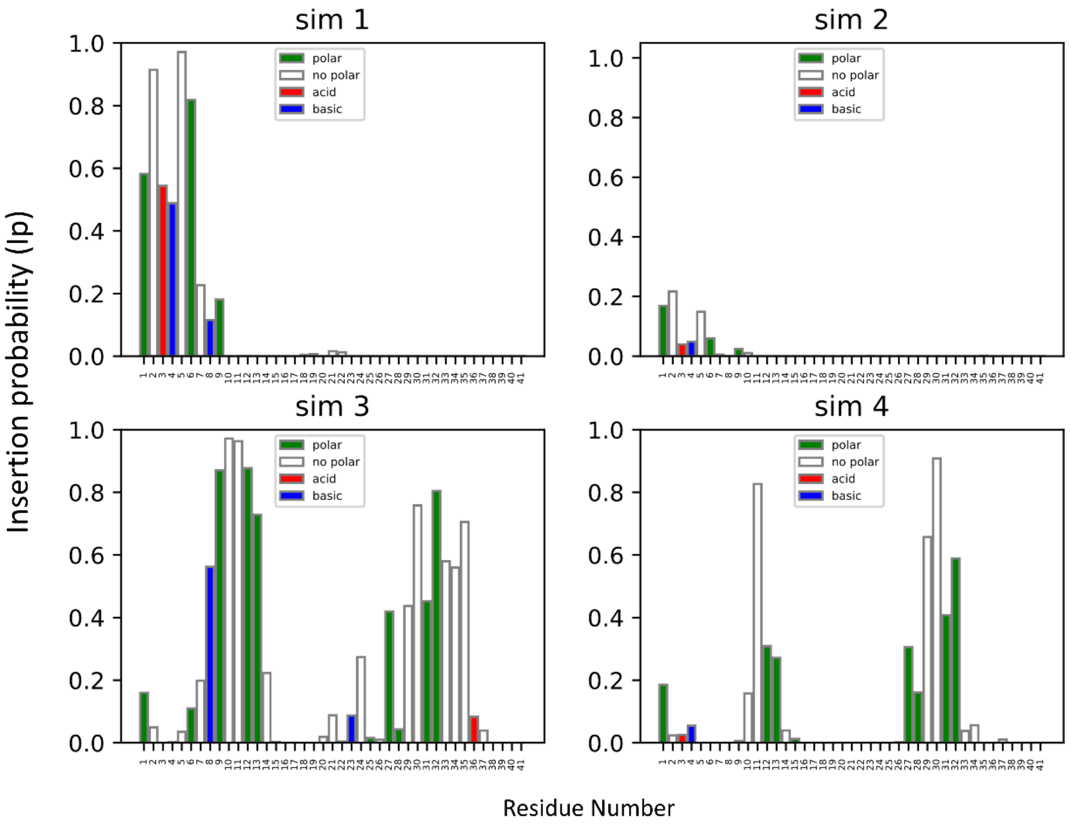

Figure S10. Insertions probability for each all-atom simulation of SNX-482 in POPC: POPG (1:3)

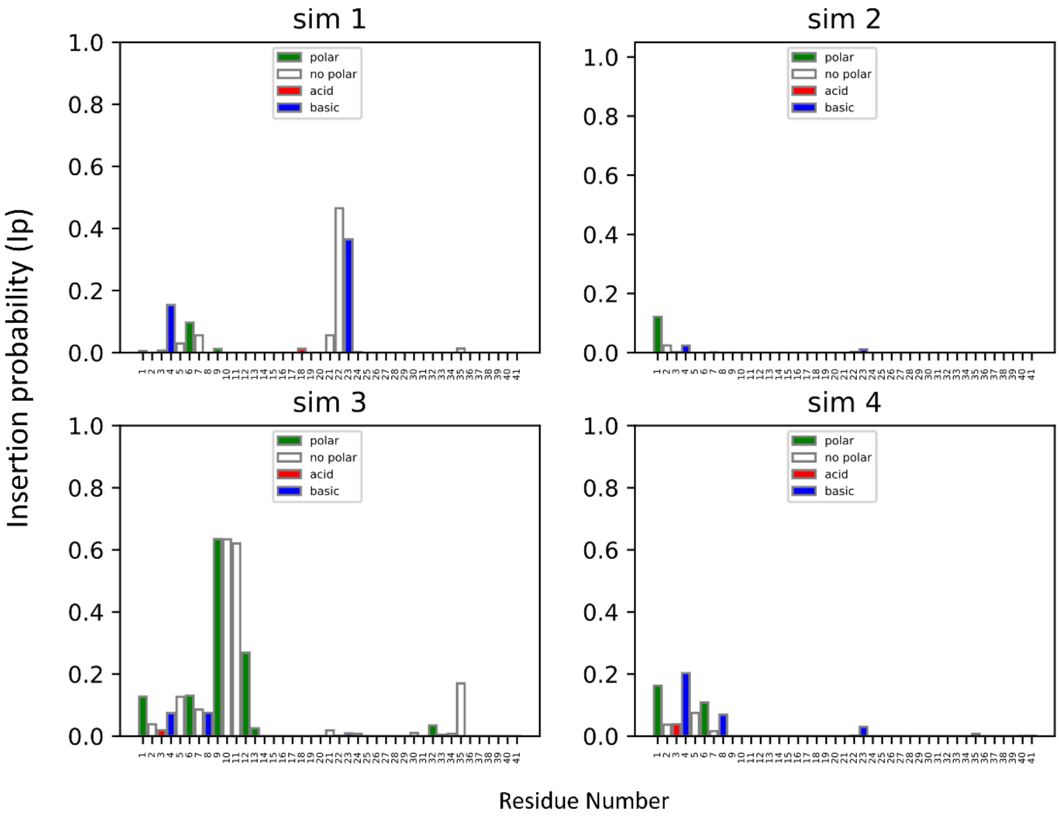

Figure S11. Insertions probability for each all-atom simulation of SNX-482 in POPG

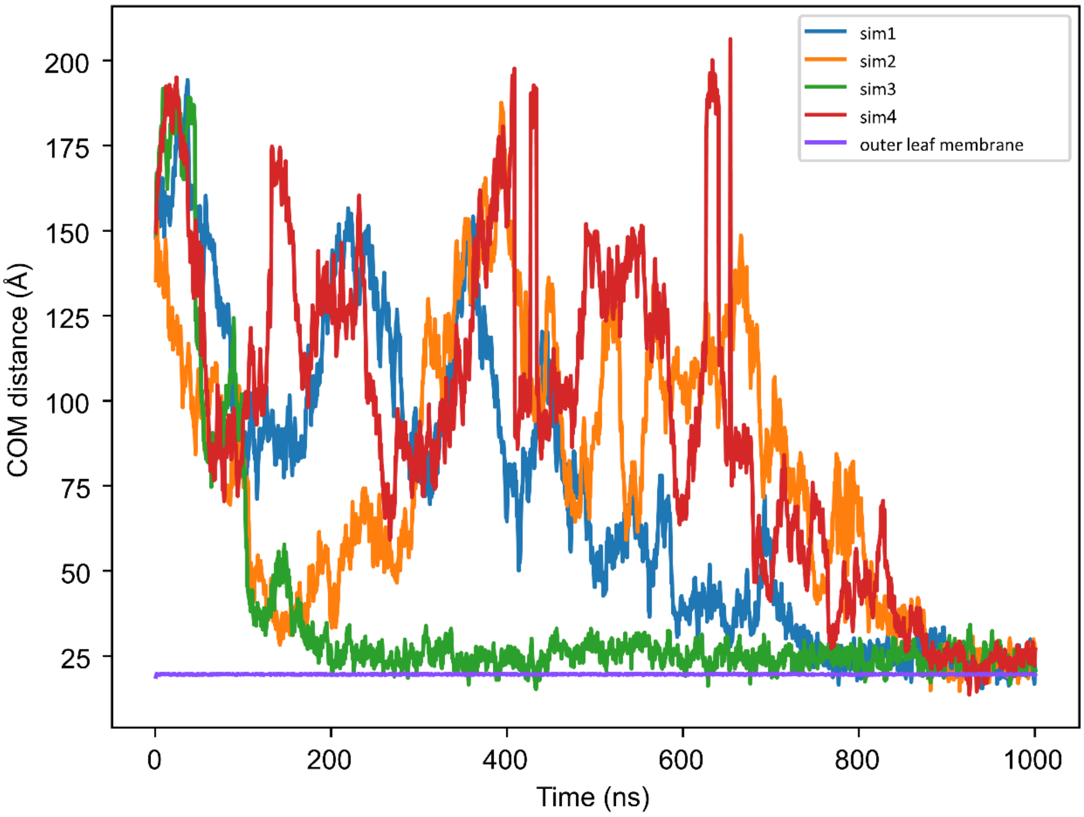

Figure S12. COM distance between toxin and POPC: POPG (1:1) membrane in CG simulations. The purple line is the distance from the center of mass of the membrane to the outer leaf membrane.

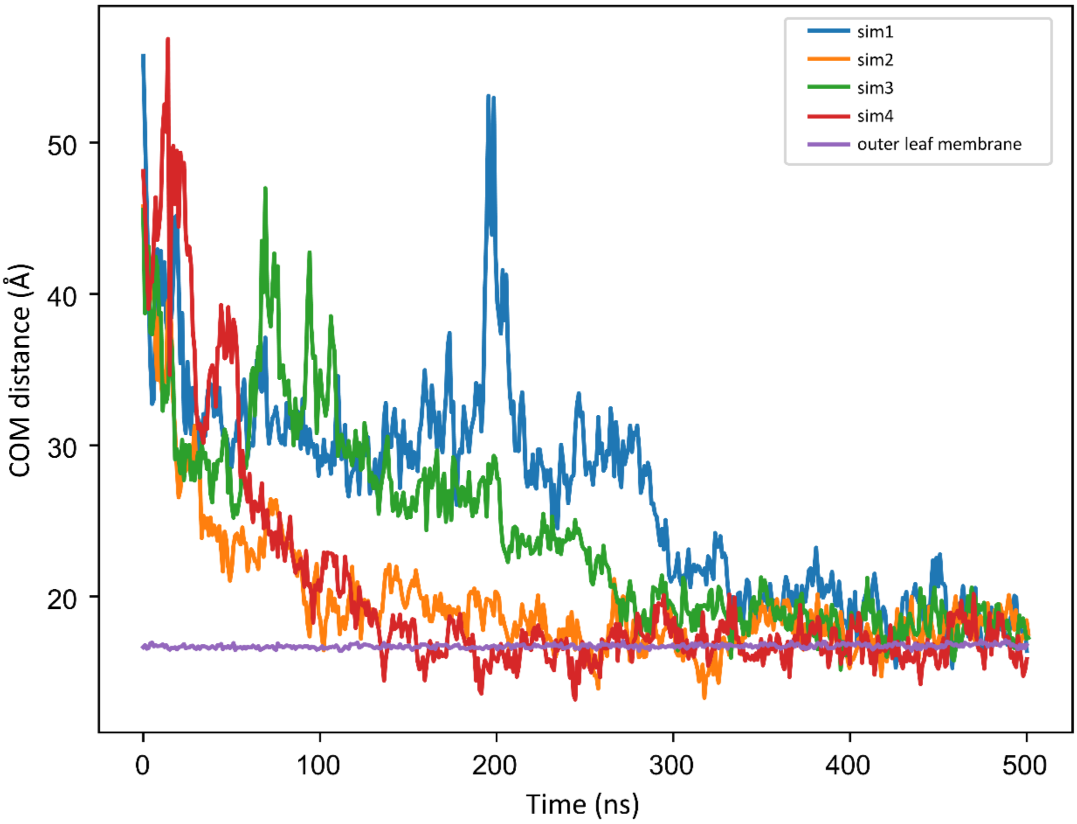

Figure S13. COM distance between toxin and POPC: POPG (3:1) membrane in AT simulations. The purple line is the distance from the center of mass of the membrane to the outer leaf membrane.

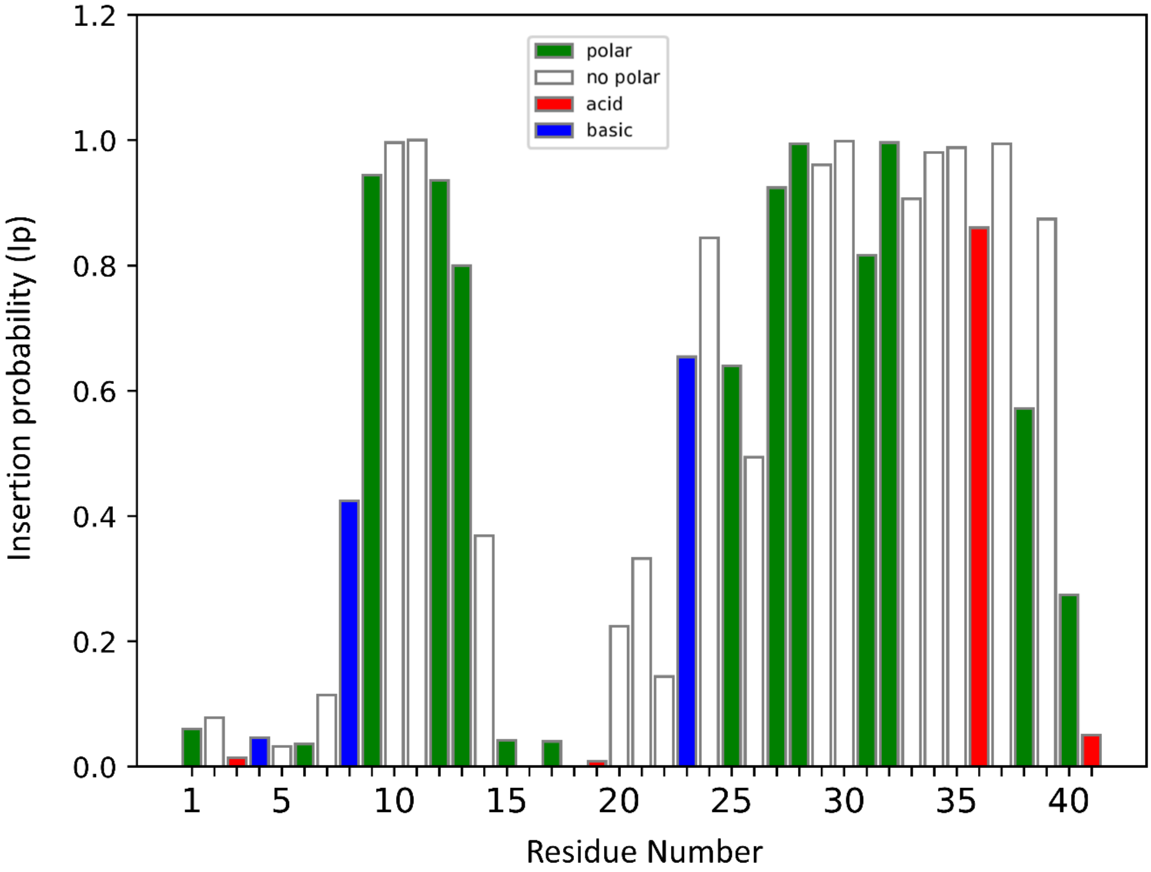

Figure S14. Insertions probability computed in the last 0.5 μs of AT simulation (1.5 μs -2 μs) of SNX-482 and POPC: POPG (3: 1).

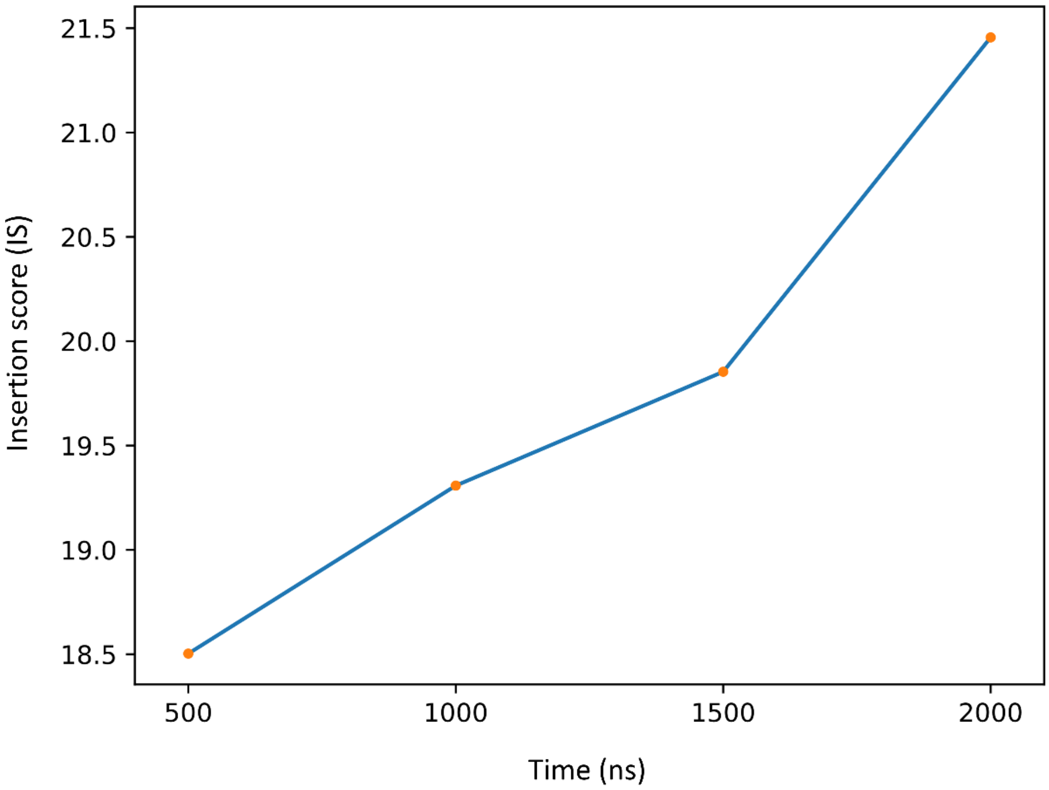

Figure S15. Insertions score computed for every 0.5 μs of SNX-482 in POPC: POPG (3: 1). during 2 μs AT simulation..

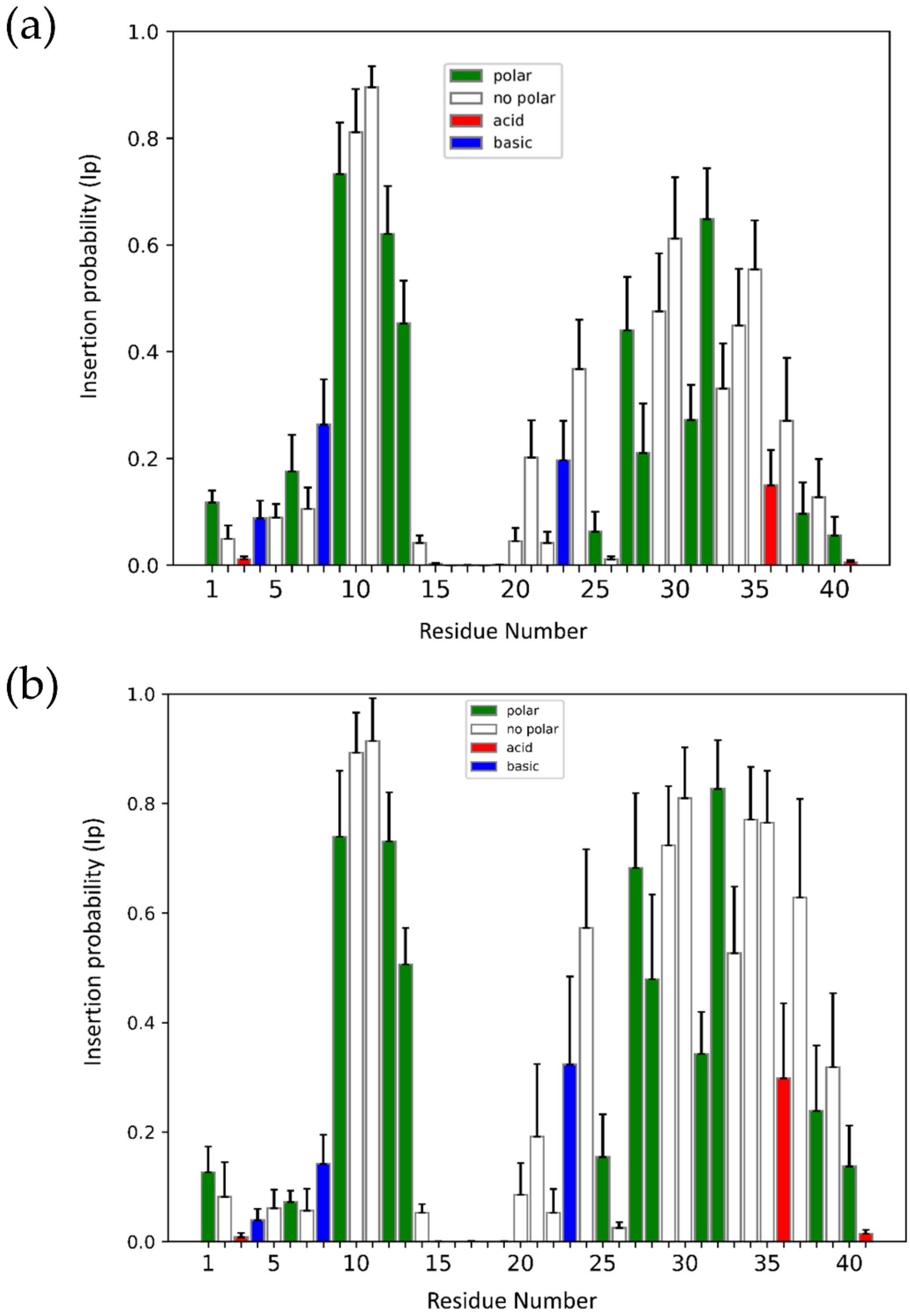

Figure S16. Sidechain insertion probability computed **(A)** a for all AT simulations showing toxin insertion (n=10) or **(B)** for all MD on POPC: POPG (3: 1) membrane.

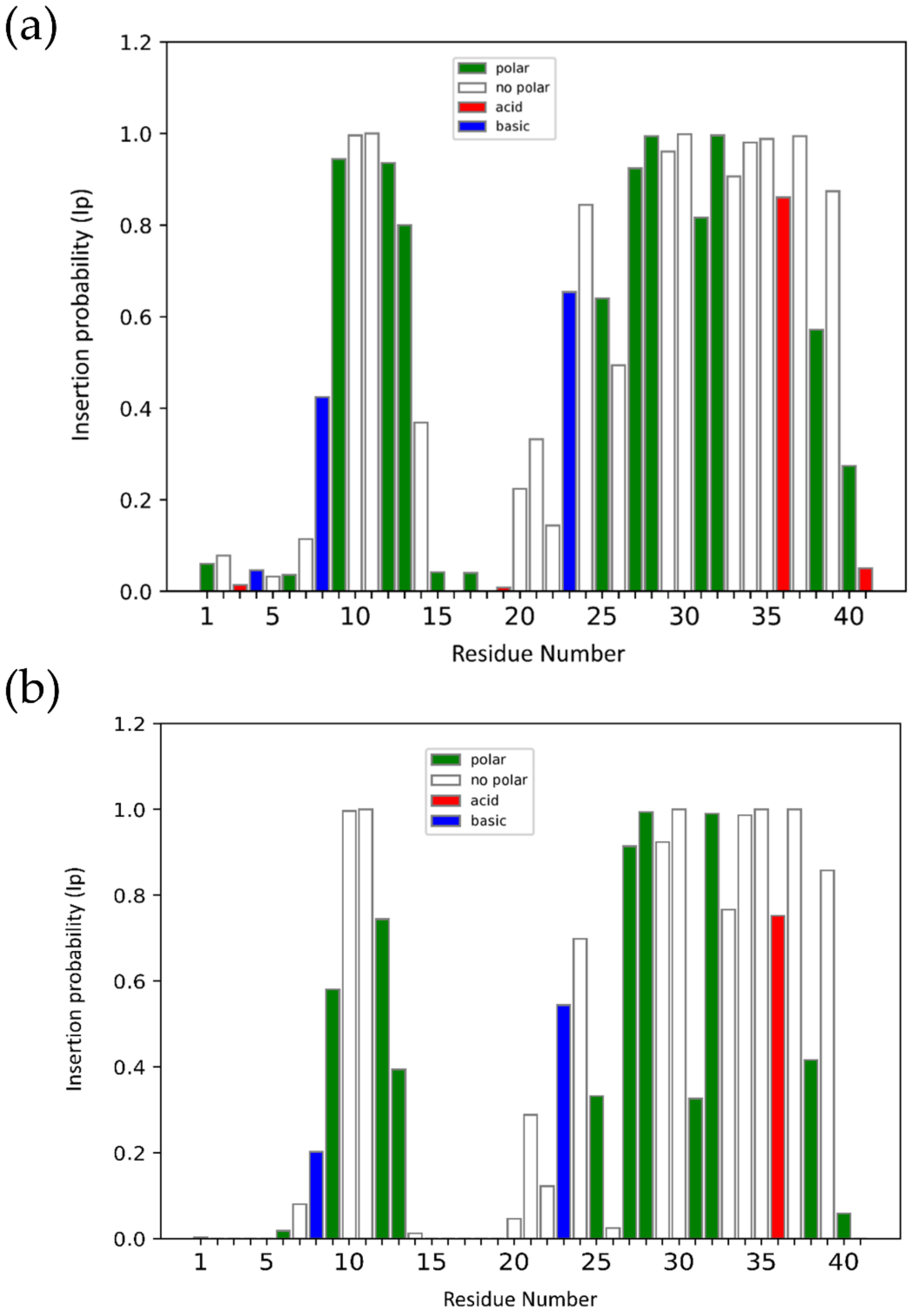

Figure S17. Insertions probability computed in the last 0.5 μs of AT simulation (1.5 μs -2 μs) of SNX-482 and POPC: POPG (3: 1) long simulation**. A)** Ip, when residues that fall below the phospholipid's head are shown in A. **B)** Ip considering residues laid below the first carbon of the fatty acid chain.

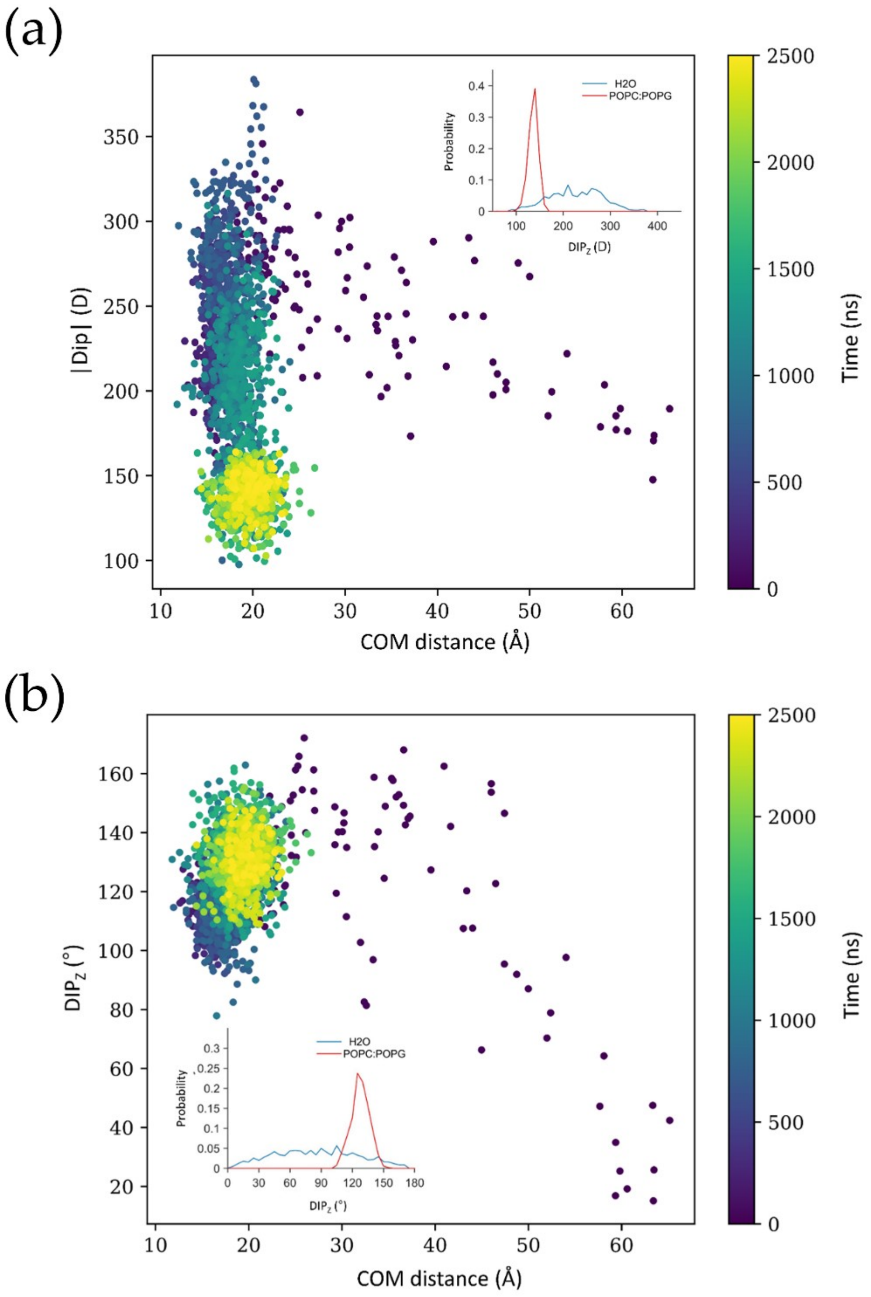

Figure S18. Dipole magnitude (A) and angle (B) of SNX-482 in POPC: POPG (3:1) during 2.5 μ AT simulation as a function of COM distance between the membrane and the toxin. Each point is colored according to the simulation time.

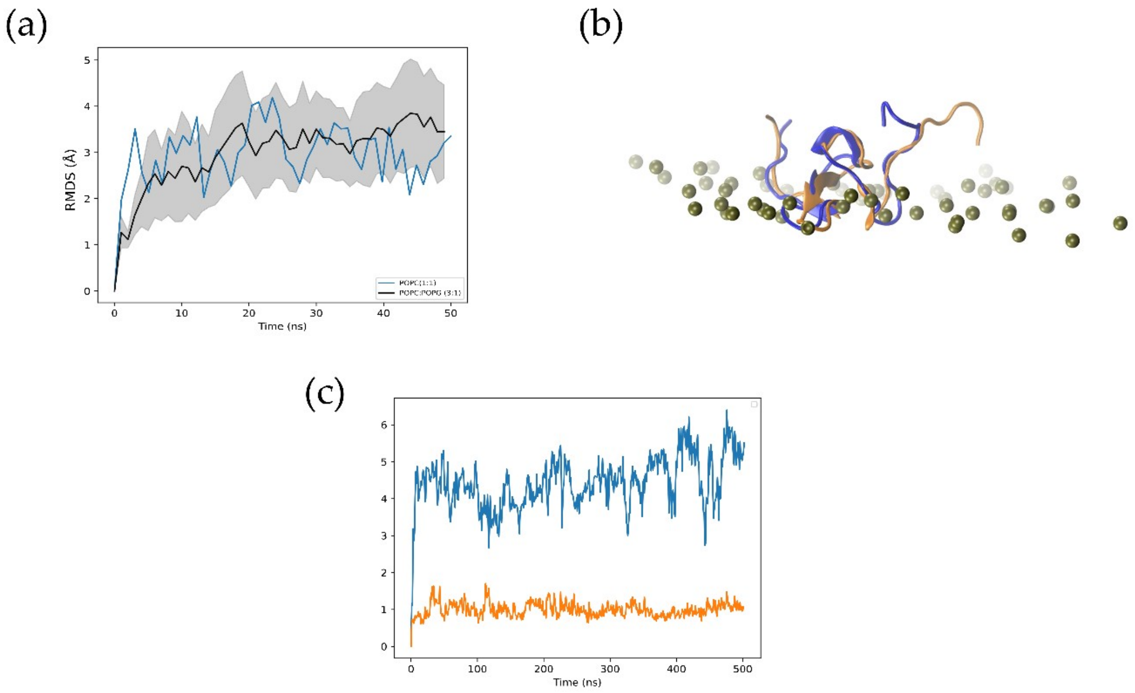

Figure S19. A) SNX-482 α-carbon RMSD during the final 0.5 μs of the 2 μs AT simulation of 3:1 system (toxin-bound, orange line) and the toxin in solution (blue line). **B)** Aligned representative structures of the membrane-bound toxin for the 3:1 (orange) and 1:0 system (blue). α-carbon RMSD of inserted residues (9-14 and 30-34) is 1.5. **C)** RMDS of 1:0 (blue; n=1) and 3:1 system (black; n=4). Standard deviation for system 3:1 is shown as a shaded area.

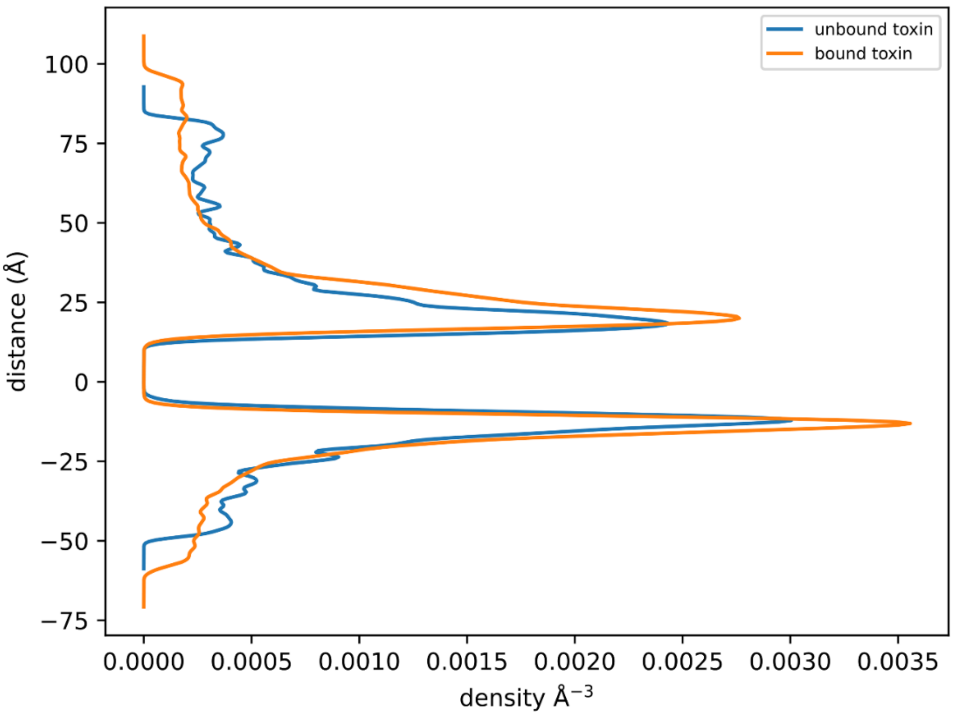

Figure S20. Ion number densities along the z for the 3:1 system for the bound (blue) and unbound states.

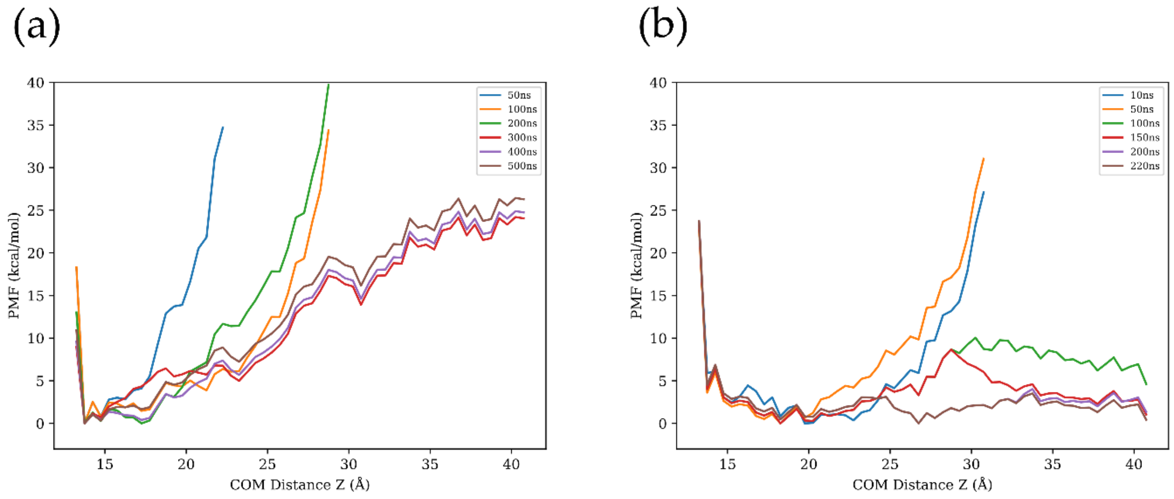

Figure S21. PMF profiles at different calculation times for 3:1 (a) and 1:0 (b) systems.
